# HyperFoods: Machine intelligent mapping of cancer-beating molecules in foods

**DOI:** 10.1038/s41598-019-45349-y

**Published:** 2019-07-03

**Authors:** Kirill Veselkov, Guadalupe Gonzalez, Shahad Aljifri, Dieter Galea, Reza Mirnezami, Jozef Youssef, Michael Bronstein, Ivan Laponogov

**Affiliations:** 1 Department of Surgery and Cancer, Faculty of Medicine, Imperial College London, London, SW7 2AZ UK; 2 Kitchen Theory, London, EN5 4LG UK; 3 Department of Computing, Faculty of Engineering, Imperial College London, London, SW7 2AZ UK

**Keywords:** Machine learning, Systems analysis, Nutrition

## Abstract

Recent data indicate that up-to 30–40% of cancers can be prevented by dietary and lifestyle measures alone. Herein, we introduce a unique network-based machine learning platform to identify putative food-based cancer-beating molecules. These have been identified through their molecular biological network commonality with clinically approved anti-cancer therapies. A machine-learning algorithm of random walks on graphs (operating within the supercomputing DreamLab platform) was used to simulate drug actions on human interactome networks to obtain genome-wide activity profiles of 1962 approved drugs (199 of which were classified as “anti-cancer” with their primary indications). A supervised approach was employed to predict cancer-beating molecules using these ‘learned’ interactome activity profiles. The validated model performance predicted anti-cancer therapeutics with classification accuracy of 84–90%. A comprehensive database of 7962 bioactive molecules within foods was fed into the model, which predicted 110 cancer-beating molecules (defined by anti-cancer drug likeness threshold of >70%) with expected capacity comparable to clinically approved anti-cancer drugs from a variety of chemical classes including flavonoids, terpenoids, and polyphenols. This in turn was used to construct a ‘food map’ with anti-cancer potential of each ingredient defined by the number of cancer-beating molecules found therein. Our analysis underpins the design of next-generation cancer preventative and therapeutic nutrition strategies.

## Introduction

With rapidly ageing populations, the world is experiencing an unsustainable healthcare and economic burden from chronic diseases such as cancer, cardiovascular, metabolic and neurodegenerative disorders^1,2^. Diet and nutritional factors play an essential role in the prevention of these diseases and significantly influence disease outcome in patients during and after therapy^3,4^. According to most recent data, up to 30–40% of all cancers can be prevented by dietary and lifestyle modifications alone^5,6^. Plant-based foods (*i.e*. derived from fruits and vegetables) are particularly rich in cancer-beating molecules (CBM) such as polyphenols, flavonoids, terpenoids and botanical polysaccharides^7^. Evidence from experimental studies has implicated multiple mechanisms of action by which dietary agents contribute to the prevention or treatment of various cancers^8,9^. These include regulating the activity of inflammatory mediators and growth factors, suppressing cancer cell survival, proliferation, and invasion, as well as angiogenesis and metastasis^10^.

Being able to first identify food ingredients and later design “hyperfoods” that are richest in CBMs and having health promoting or therapeutic influence, represents an unprecedented opportunity to reduce healthcare costs and potentially enhance health outcomes for chronic diseases such as cancer^11^. Since in the modern era of designer gastronomy the consumers are increasingly discerning and demanding, the design of hyperfoods is a multi-faceted optimization problem taking into account not only pro-health benefits, but also considering various aesthetic (e.g. color, texture) and sensory (*e.g*. taste, mouthfeel) characteristics. We argue that at least some parts of such design could be performed computationally, by exploiting artificial intelligence (AI) technology. As outlined in our recently published 10-point manifesto (‘The Future of Computing and Food’), this will require a collaborative approach of multiple stakeholders including food producers, chefs, designers, engineers, data scientists, sensory scientists and clinicians^12^.

The human diet contains thousands of bioactive molecules which modulate a variety of metabolic and signaling processes, drug actions, and interactions with gut microbiota in health and disease^13,14^. Investigating the influence of a single biochemical food constituent takes months to years of experimental research. Moreover, current approaches to identify active compounds within food that influence health are incapable of taking into consideration the myriad of complicating factors such as where the food comes from, how it has been cultivated, stored, processed and prepared, not to mention cooking parameters and the effect of ingredient combinations. Given the vast molecular space, predictive identification of bioactive compounds for tailored nutritional strategies using current experimental research methods is therefore not feasible. However, recent advances in AI technologies coupled with the explosive growth of large-scale multi-source (“-omics”) data on food, drugs and diseases offers a unique opportunity to identify molecules within foods to potentially prevent and/or fight disease phenotypes^15,16^. These studies have identified molecules within foods based on either structural similarity or the similarity of individual gene-encoding protein targets to those of approved therapeutics. However, even minor change in the chemical structure of a molecule can lead to drastically different biological outcomes, and complex diseases, such as cancer, cannot be explained by deregulated activity of individual genes/proteins. Several recent computational studies have attempted to leverage “-*omics*” data to extract insights on positive and/or adverse interactions between foods, drugs and disease. Zheng *et al*. used publicly available gene expression and interactome data of cell cultures and animal models to identify drugs and diets anti-correlated with disease gene expression phenotypes^17^. Due to the small size of existing diet-induced gene expression datasets, this correlation-driven analysis was restricted to a very limited number of foods. Nevertheless, intriguing diet-disease associations have been identified through this approach. A combined chemo-informatics and text mining strategy was applied to several million PubMed abstracts to define health-promoting or detrimental associations between the molecular constituents of plant-based foods and disease phenotypes^16,18^. This strategy was subsequently extended to identify food components interfering with drug metabolizing enzymes (“pharmacokinetics”) or interacting with drug targets (“pharmacodynamics”)^17^. Although of great promise, the automated relation extraction systems based on natural language processing (NLP) have thus far been tested on a very small subset (<200) of somewhat subjectively annotated abstracts. As we highlighted recently, their application at the scale of multi-million article databases such as PubMed warrants extensive validation of the rate of false discoveries and extraction of supporting evidence to build trust in the computer-derived associations^19^. Nevertheless, these developments have been instrumental to the compilation of “-omics” food databases and public repositories such as FooDB, FlavorDB and NutriChem^15,16,18^.

Complex diseases such as cancer cannot be explained by single gene defects but rather involves a breakdown of various molecular functions mediated through a set of molecular interactions (“networks”)^20–22^. The diversity of the resulting cancer molecular phenotypes makes it very difficult to identify specific molecular targets for cancer prevention or treatment. We hypothesize that an effective cancer preventative or therapeutic intervention should target multiple biochemical pathways implicated in carcinogenesis such as inflammation, cell proliferation, cell cycle, apoptosis and angiogenesis. In line with this hypothesis, we have tailored a machine–learning based strategy that predicts CBMs based on “learned” molecular networks targeted by clinically validated anti-cancer therapies. Our strategy includes the combined use of unsupervised learning on graphs to simulate the downstream influence of therapeutics on human proteome networks (from “sparse” protein target datasets) followed by supervised learning to identify predictive (sub-)networks for CBMs. Model performance was assessed using a 10-fold cross-validation strategy, which confirmed accurate prediction of anti-cancer therapeutics. A comprehensive database of 7692 bioactive molecules within foods was fed into the model to predict ~110 CBMs, resulting in a compiled list of hyperfoods exhibiting the largest number of potential CBMs (ACL > 0.7). Furthermore, the developed approach can be easily extrapolated in the future to cover other types of diseases (e.g. diabetes) and health issues to provide a comprehensive multi-faceted picture of health-promoting food molecules and optimize existing cooking recipes for the maximally positive health impact. We envisage that this first list of “cancer-beating” foods will serve as one of the pillars in the foundation for the future of gastronomic medicine and should aid the creation of personalized “food passports” to provide nutritious, tailored and therapeutically functional foods for the population. However, significant future work will be required to validate and quantify the therapeutic effects of these proposed hyperfoods as well as optimize cultivation, storage, processing and cooking parameters of their ingredients.

## Results and Discussion

### Network-based machine-learning strategy for drug and food repositioning

**Figure 1:**
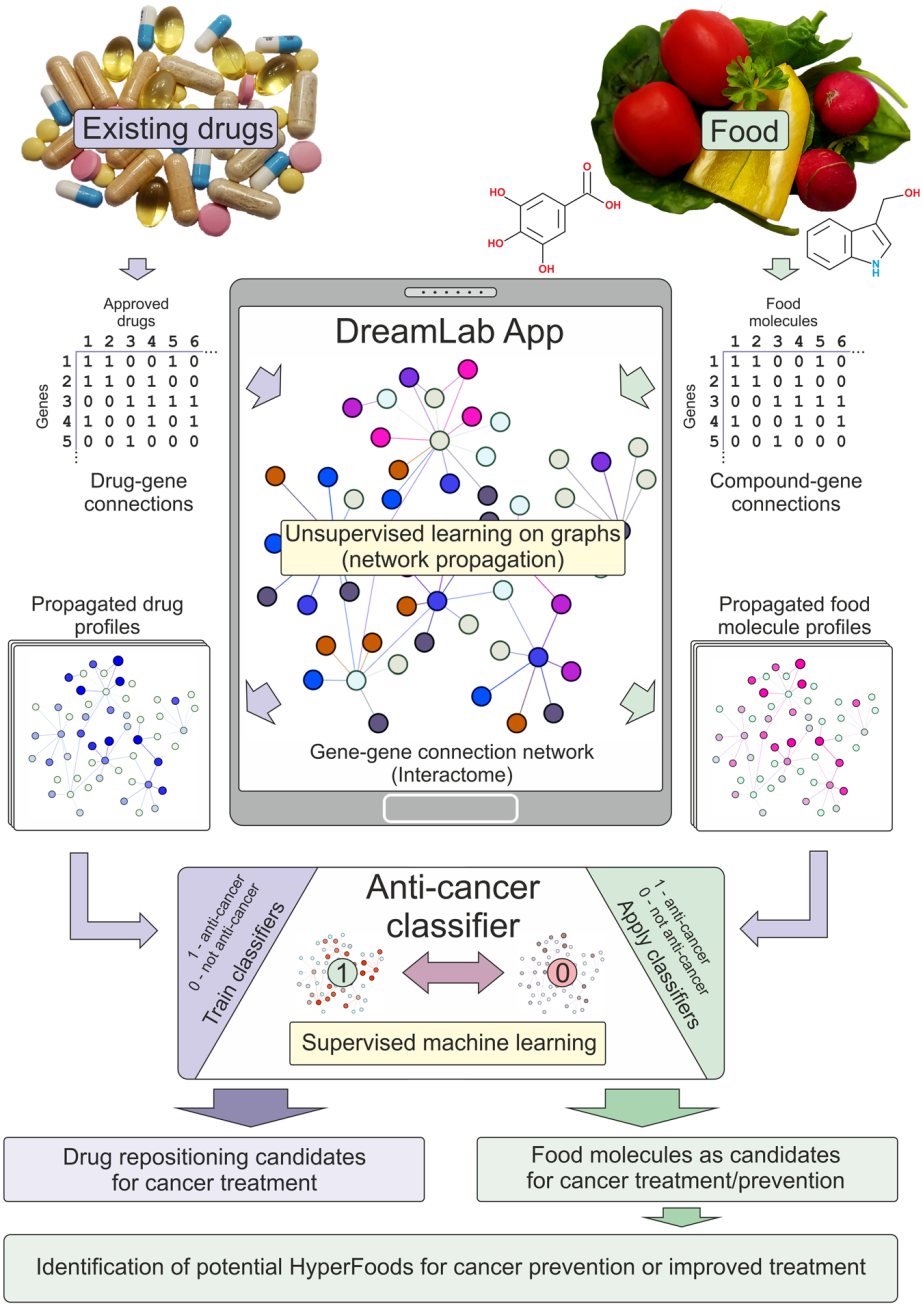
Schematic diagram of the overall workflow.

The work presented herein exploits publicly available data on molecule to gene-encoded protein interactions as well as protein-protein interaction data. In brief, the sparse data of interactions between drugs and their protein/gene targets are initially mapped on large-scale interactome networks - a whole set of protein-to-protein interactions in humans (here and further due to the specifics of the existing interaction datasets, “gene” and “protein” terms can be used interchangeably). Most drugs exert their biomedical and functional activity by binding to a specific subset of proteins. Proteins rarely function in isolation but rather operate as part of highly interconnected networks^23^. Taking this into account, we have tailored random walks on graphs with restarts (controlled by a single network diffusion parameter “*c*”) to simulate the perturbation of individual drugs on human proteome networks using aggregated datasets of their targeted proteins. Similar network-based propagation approaches have been recently compared favourably to predict drug-target interactions, and evaluate network perturbations caused by cancer mutations for improved patient stratification^24,25^. This network diffusion transforms a short list of proteins targeted by a given molecule/drug into a genome-wide profile of gene scores based on their network proximity to target candidates. Using the genome-wide profiles of drugs, the supervised machine-learning strategy (“maximum margin criterion” and support vector machines, in this case) is trained to accurately classify “anti-cancer” (vs “other”) properties of molecules. The best obtained models were used to predict the probability of a given existing approved drug to exhibit anti-cancer properties. After validation of the predictive capacity of the model for anti-cancer drug repositioning, the same machine learning strategy was applied to predict various cancer-beating molecules within foods (Fig. 1). It should be noted that there are various methodologies for drug repositioning such as molecular structural commonality, molecular target similarity as well as shared genetic or phenotypic (e.g. side effect profile) influence^26,27^. However, these approaches mandate additional data sets (such as gene-expression data, proteomics, metabolomics or phenotypic effect data) for model building. In the search for food-based cancer beating molecules, these data are very limited.

### Benchmarking and optimization of machine learning strategy

Among the machine learning methods tried, MMC (maximum margin criteria)^28^ and SVM^29^ with linear kernel showed comparable performance and relatively good processing speed (including parameter optimization, model training and prediction on 10-fold cross-validation). Radial kernel SVM did not exceed the performance of the linear methods and at the same time required much longer processing time (the best radial kernel SVM F1-score achieved is of 0.85 vs 0.86 for linear kernel SVM). Furthermore, the optimal gamma parameter for the radial SVMs tends to be very low (~10^−7^), effectively making them similar to the linear kernel SVMs. We have also explored 2 neural network classifiers and 2 regularized LASSO/Elastic Net logistic classifiers to see whether they bring any improvement in the classification accuracy. For the best performing type of interactome and settings of random walk on graphs, these more advanced approaches resulted in prediction accuracies comparable to linear SVM and MMC (see SI Appendix M1). This is well known in genomics studies involving a small number of examples and a large number of features, where the linear classifiers are preferred because of their transparency and biological interpretability. As a result, the major focus was made on linear kernel SVM and MMC methods for the final round of optimization. The best F-score achievable was of 0.86 with linear kernel SVM with 84% correct anti-cancer predictions and 90% correct non-anticancer predictions (see SI Dataset S1). Re-running the optimization multiple times for the same settings showed consistent performance (maximum 1–2% difference). Based on these results, it was decided to select the top 700 models (F-score >  = 0.84) for anti-cancer likeness prediction from models based on linear kernel SVM and MMC for existing approved drugs (SI Dataset S2) and food compounds (SI Dataset S3). Interestingly, *log-*transformation of the input propagated profiles was systematically shown to increase performance of the classifiers. This is likely because some individual isolated genes, which do not propagate and thus stay with a very high perturbation level would have lesser effect on the overall profile in log-space. At the same time “*c*” parameter of the random walker and different matching settings between compounds and genes had less pronounced effects. Gene-gene connection thresholds were also not strongly influential except in the case of BioPlex interactome. This is likely because connections provided by STRING tend to include a wide range of knowledge sources giving a more representative and complete graph of gene-gene (or protein-protein) interactions and the sheer number of connections can compensate for the larger values of “*c*” and higher thresholds used. We have also evaluated individual gene influence on the final classification, *i.e*. gene importance, by finding the correlation between the gene levels and the prediction outcomes for the optimized model. The full table of averaged importance predictions for the top selected 700 models is provided as SI Dataset S4. As expected, the top-rated genes are involved in cell proliferation control and their mutations are often associated with cancer. This provides transparency to the machine learning based prediction of anti-cancer properties of the drugs.

### Pathway analytics and differential interactome

**Figure 2:**
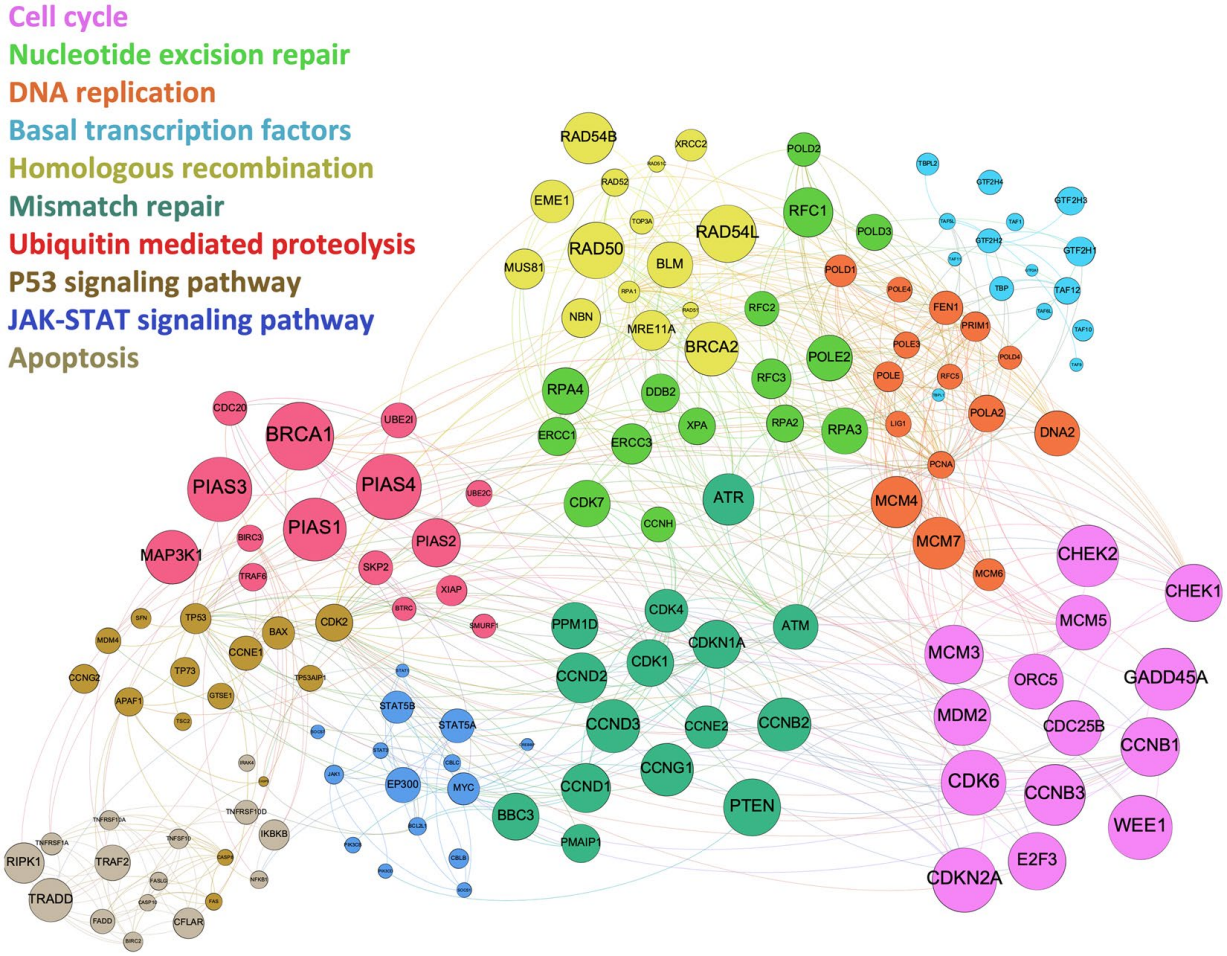
Relevant genes and pathways derived from machine learning models for prediction of anti-cancer therapeutics tested in human trials. Individual node size corresponds to the relative discriminating capacity of a given gene-encoded protein and node color illustrates shared biological pathway functionality.

A list of the most influential genes/proteins for predicting anti-cancer therapeutics derived from network-based machine learning was subjected to pathway analytics using gene-set enrichment (SI Dataset S4). Among the top 25 impacted pathways were cell cycle, DNA replication, apoptosis, *p*-53 signaling, JAK-STAT signaling and mismatch repair as well as various cancer-specific pathways. It adds to the biological plausibility of the modelling approach used here that the pathways identified as key drivers are those consistently implicated in cancer development and progression. In Fig. 2, relevant discriminating genes and their corresponding impacted pathways are presented. Here, individual node size corresponds to the relative discriminating capacity of a given gene-encoded protein and node color illustrates shared biological pathway functionality. Increasingly, it is understood that the mechanistic bases for cancer cell survival, dissemination and therapeutic resistance are manifold and involve multiple biochemical pathways. Most machine-learning derived pathways in our analysis have been suggested as targets for cancer prevention or therapeutic interventions^30–32^. Therefore, the “ideal” anti-cancer agent should be capable of disrupting multiple pro-tumorigenic biochemical processes. The machine learning approach presented here highlights the biological pathways influenced by currently utilized anti-cancer therapeutics, and thus permits in parallel a targeted search for unique agents, in this case bioactive compounds with foods, with the potential to impact on multiple pathways simultaneously.

### Drug repositioning in cancer using interactomics

The full prediction summary is presented in SI Dataset S2. As expected most compounds currently in use as cancer therapeutics demonstrated strong anti-cancer probability. Interestingly, several compounds which are not conventionally used in cancer treatment demonstrated very high anti-cancer likeness (ACL). The available literature on these compounds was further interrogated to understand the mechanistic basis for the potential anticancer effect(s) of these agents. For example, quinolone-derivative rosoxacin and quinoline-based clioquinol primary act as anti-microbial and anti-fungal agents, respectively. However, the analysis presented here indicates a potential direct role for these therapeutics in cancer. The quinolone antibiotics were shown to have a significant inhibiting potency against eukaryotic topoisomerase-II resulting in cytotoxicity of various cancer cell types^33^. This group of compounds can be explored in comparison to human topoisomerase-II inhibiting anti-tumor drugs such as doxorubicin and etoposide. Clioquinol is a chelator of zinc, copper and iron which are known to be involved in both carcinogenesis and angiogenesis^34^. The anti-neoplastic activity of clioquinol is thought to be through several potential mechanisms including NF-kB apoptosis induction, mTOR signaling and inhibition of lysosome^35^. Although of great promise its role in cancer therapy remains largely unexplored in clinical settings. The anti-diabetic drugs such as metformin and chromium picolinate, also emerged as potential candidates for anti-cancer drug repositioning from this evaluation. The molecular mechanisms responsible for this association remain uncertain, however both agents are used to alleviate insulin resistance through modulation of the insulin signaling cascade, and a number of studies have shown that chromium specifically alters proximal insulin signaling and directly effects insulin receptor phosphorylation and kinase activity^36^. The downstream consequences of therapy with both metformin and chromium is the reduction in insulin and insulin-like growth factor levels, which in turn is understood to inhibit several key processes within the mTOR signaling pathway, which is a central molecular driver of a variety of cancers^37^. Correspondingly a strong association has been shown on pooled analysis between metformin usage and incidence of cancer in type II diabetics^37^. By contrast, the chromium picolinate might act as a double “edged sword” due to its capacity to interfere with DNA leading to structural genetic lesions and thereby promoting carcinogenesis^38^. This example highlights the limitation of our approach to identify molecules that interact with relevant carcinogenetic processes irrespective of the nature of the interaction (*i.e*. inhibition or stimulation). Identifying the nature of molecular interactions would require additional datasets such as gene expression or proteomics but these are not generally available in the case of food-based molecules.

### Prediction of cancer-beating molecules in foods

**Figure 3:**
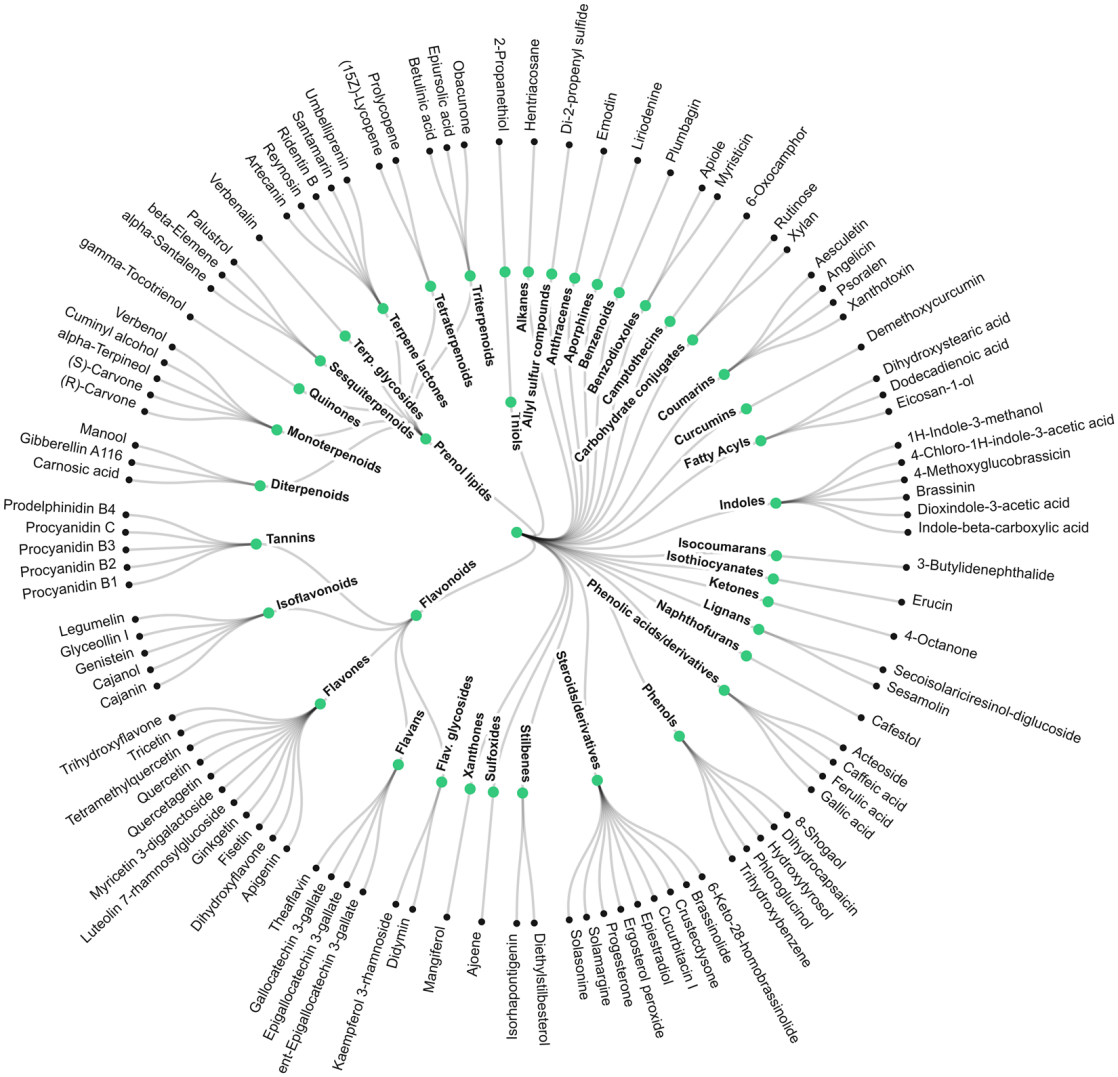
Hierarchical classification of the top 110 predicted cancer-beating molecules in food with anti-cancer drug likeness of >0.7.

**Figure 4:**
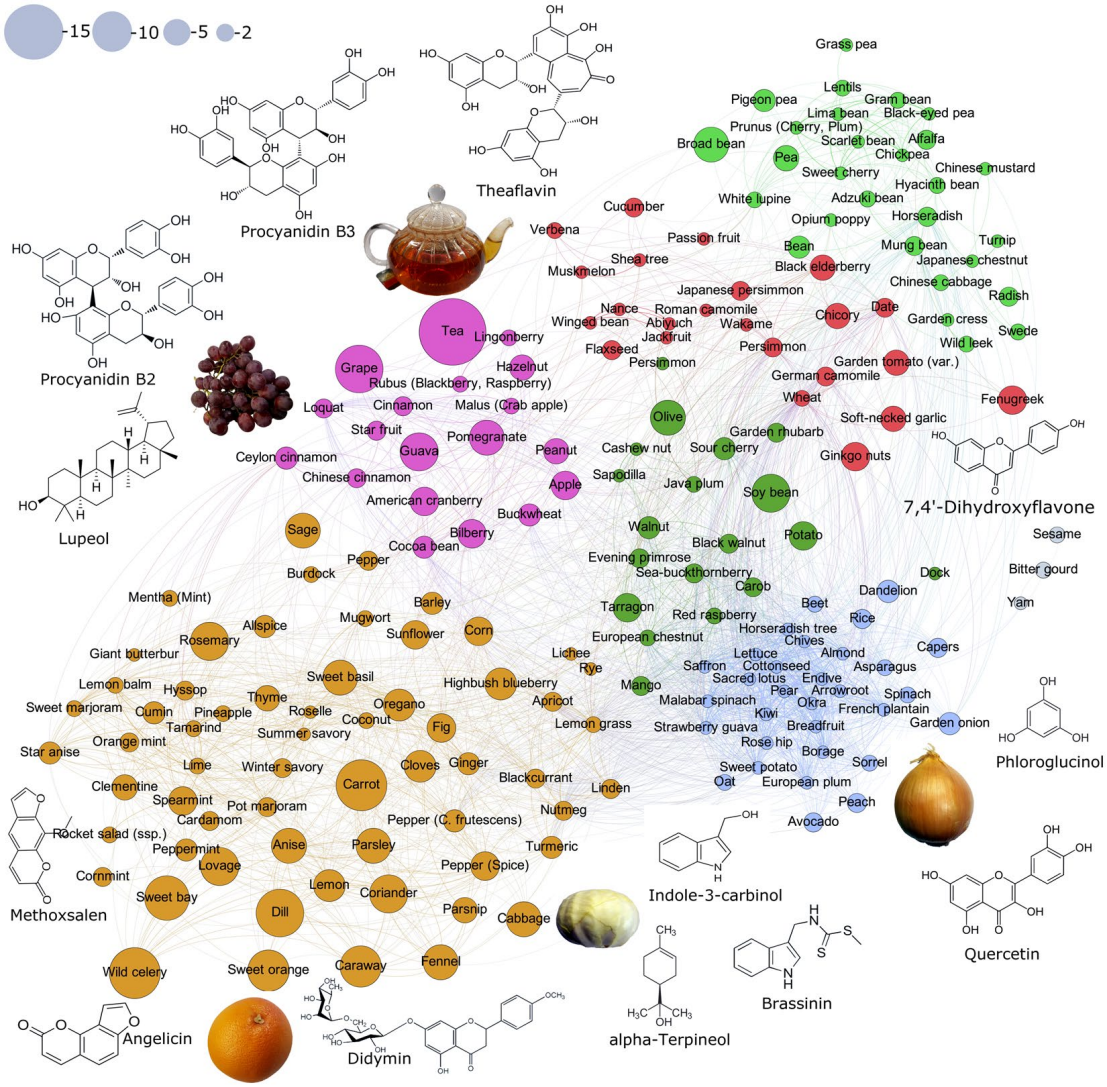
The contained profiles of compounds within selective foods, which were highly likely to be effective in fighting cancer. Each node in the figure denotes a particular food item and node size in each case is proportional to the number of CBMs. The link between nodes reflects the pairwise correlation profile of CBMs in foods, thus the clusters of foods illustrate molecular commonality between them.

From all small molecules approved for anti-cancer therapies, almost half are derived from natural products^39^. These drugs are generally more tolerated and less toxic to normal cells^39^. The methodology outlined above was next applied to predicting the anti-cancer likeness of ~7692 bioactive compounds across various food categories. Here a comprehensive view of drug-like molecules in food is provided, unlike most studies in the literature to date which have tended to focus on a single compound or a single food type. Approximately 110 molecules from different chemical classes (see Fig. 3), including terpenoids, isoflavonoids, flavonoids, poly-phenols and brosso-steroids were identified and mapped according to their food sources using multiple experimental databases. A complete list of food molecules ranked by proxy according to anti-cancer drug likeness of >0.1 is provided in SI Dataset S3. Using the unsupervised learning random walk on graphs, we have propagated the influence of the most promising molecules on human interactome networks and identified their impacted molecular pathways (for detailed analysis see SI Dataset S3 and SI Dataset S5 only for compounds with ACL > 0.7). SI Appendix Table S1 summarizes a list of cancer-beating compounds identified in the present study with high ACL > 0.7 and their associated food sources. Furthermore, we have conducted a comprehensive review of the available literature on the top anti-cancer drug like molecules (with ACL > 0.9) and their putative molecular mechanisms of anti-cancer actions (SI Appendix Table S2). Both computational analysis and experimental data from literature show that the pathways and mechanisms responsible for these anti-cancer properties cover the breadth of our current understanding of the multi-step process of carcinogenesis. These include anti-inflammatory, pro-apoptotic effects, potent antioxidant activity and scavenging free radicals; regulation of gene expression in cell proliferation, cell differentiation, oncogenes, and tumor suppressor genes; modulation of enzyme activities in detoxification, oxidation, regulation of hormone metabolism; and antibacterial and antiviral effects^40^. For example, 3-indole-carbinol, which is found abundantly in members of the Brassica oleracrea family of vegetables (including cabbage, broccoli and brussel sprout) appears to be one of the most strongly anti-cancer-like molecules. This bioactive compound has been shown to target multiple aspects of cancer cell cycle regulation and survival, including caspase activation, oestrogen metabolism and receptor signaling and endoplasmic reticulum function (see SI Appendix Table S2 and reference therein). Other prominent examples include dydamin, which is a flavonoid glycoside found in citrus fruits and apigenin, which is particularly abundant in coriander, parsley and dill. Both are understood to influence apoptotic pathways as well as cell cycle arrest mechanisms and are believed to suppress cancer cell migration and invasion (see SI Appendix Table S2 and reference therein). Figure 4 provides a visual summary of CBMs associated with strong anti-cancer likeness. Each node in the figure denotes a particular food item and node size in each case is proportional to the number of CBMs. The link between nodes reflects the pairwise correlation profile of CBMs in foods, thus the clusters of foods seen in Fig. 4 illustrate molecular commonality between them. The foods that show greatest diversity in CBMs include tea, grape, carrot, coriander, sweet orange, dill, cabbage and wild celery.

### Food map and phytochemical synergy

The potential of food sources to exert their preventative or therapeutic capacity depends upon the bioavailability and diversity of disease-beating molecular compounds contained therein^41^. A key limitation in regards to the existing literature on food-based compounds is the largely one-dimensional view that is commonly taken, with studies tending to focus on specific molecular components in isolation, for example anti-oxidants^40^. It is accepted that regular consumption of fruits and vegetables can reduce the risk of carcinogenesis (42). However, when antiproliferative agents acting in isolation have been subjected to clinical trial evaluation they do not appear to consistently confer the same level of benefit. The point is simply illustrated in the case of the apple; apple extracts contain bioactive compounds that have been shown to inhibit tumor cell growth *in vitro*. However, interestingly phytochemicals in apples with the peel preserved inhibit colon cancer cell proliferation by 43%, whereas this effect was found to be reduced to 29% when apple without peel was tested^42^. From these observations it is therefore clear that the successful implementation of food-based approaches in the fight against complex diseases such as cancer will rely on a consortium of biologically active substances, such as those present in whole fruits and vegetables, in order to increase the chances of success. The anti-cancer properties of a given food will thus be determined by (1) the additive, antagonistic and synergistic actions of their individual components and (2) the way in which these simultaneously modulate different intracellular oncogenic pathways. Both of these conditions are fulfilled in the case of tea for example, which we found to strongly exhibit anti-cancer drug-like properties compared with other food ingredients. Tea is a rich source of anti-cancer molecules from catechins (epigallocatechingallate), terpenoids (lupeol) and tannins (procyanidin) and, three of which exert strong and complementary anti-cancer effects, by protecting reactive oxidative species induced DNA damage, suppressing inflammation and inducing apoptosis and cancer cell cycle arrest, respectively. Correspondingly, several recent meta-analyses demonstrated that the consumption of green tea demonstrated delayed cancer onset, lower rates of cancer recurrence after treatment, and increased rates of long-term cancer remission^43,44^. Other examples include citrus fruits such as sweet orange, which contains dydimin (citrus flavonoid), obacunone (limonoid glucose) and β-elemene with strong anti-oxidant, pro-apoptotic and chemosensitization effects, respectively. The latter have strong effects particularly against drug-resistant and complex malignancies across different types of cancers. The inverse associations between citrus fruit intake and incidence of different types of cancers were confirmed by meta-analysis of multiple case-control and prospective observational studies^45^. With this understanding we have constructed the anti-cancer drug-like molecular profiles comprised of over 250 different food sources (see Fig. 4 and SI Appendix Table S1).

## Conclusions

Using a network-based machine learning method, we have shown that plant-based foods such as tea, carrot, celery, orange, grape, coriander, cabbage and dill contain the largest number of molecules with high anti-cancer likeness through exerting influence on molecular networks in a similar fashion to existing therapeutics. Our large scale computational analysis further demonstrates more cancer-beating potential of certain foods calling for more tailored nutritional strategies. However, it is also important to acknowledge the limitations of the proposed methodology; firstly, concentrations of bioactive molecules are not taken into account and it is unclear they would be present in sufficient enough concentration to exert their beneficial biological activity. Furthermore, the proposed methodology only accounts for interactions between bioactive food compounds and cancer-related molecular networks, without explicit regard for directionality of these relationships. In addition, the methods described here do not take into account specific cancer molecular phenotypic characteristics. Finally, drug-food interactions have not been evaluated, and it is not clear whether these will lead to synergistic or antagonistic effects where they act on common molecular networks (pharmacodynamics), or whether this combination will disrupt drug metabolism itself (pharmacokinetics). Nevertheless, food represents the single biggest modifiable aspect of an individual’s health and the machine learning strategy described here is a first step in realizing the potential role for “smart” nutritional programmes in the prevention and treatment of cancer. The outlined methodology is not restricted to cancer and will be applicable to other health conditions. Moreover, it will pave the way to the future of hyperfoods and gastronomic medicine, encouraging the introduction of personalized “food passports” to provide nutritious, tailored and therapeutically functional foods for every individual in order to benefit the wider population.

## Methods

### DRUGS/DreamLab mobile cloud supercomputing

The methodology and results presented in this manuscript were generated within the framework of the DRUGS project (Drug Repositioning Using Grids of Smartphones) run by Imperial College London in collaboration with Vodafone Foundation. The project has benefitted from the use of smartphone-based cloud supercomputing utilizing the DreamLab App. In brief, DreamLab allows a user to donate their idle smartphone computing power for use in large-scale computational tasks. With tens-to-hundreds of thousands of smartphones united into a cloud-based computational grid, one can split computational tasks into small chunks and run them in parallel. With enough contributors, the resulting performance compares to modern high performance computing clusters.

The DRUGS project uses publicly available data about gene-gene, protein-protein, drug-gene and drug-protein interactions to model systemic effects of the drugs and disease causing mutations. This allows to find promising candidates for drug repositioning and gene-tailored selection of drug combinations for treatment of different cancer types. Due to a massive number of potential combinations of drugs, cancer mutations and parameter settings, this project requires distributed computing to achieve viable speed and it fits perfectly within the specifications of the DreamLab architecture (high CPU usage, small memory footprint, no data exchange between jobs, small volumes of data transfer). The results presented in this manuscript are based on the initial data obtained within the DRUGS project with the aid of the DreamLab cloud computing platform, *i.e*. full propagated profiles of interactome impacts of different individual drugs and food compounds obtained for a wide range of settings. The predicted anti-cancer candidates are identified based only on the similarity of their full profiles to the known approved and clinically used anticancer drugs, which is established via machine learning approaches. Combinatorial analysis and gene-tailoring for personalized treatment recommendations are currently “work-in-progress” and fall outside of the scope of the present study.

### Aggregation of molecular data sets of drugs and foods

Clinically validated pharmacotherapeutic agents currently in clinical use were selected from DrugBank (open database of drugs, Nov 2017)^46^. Only drugs with FDA approval were incorporated into the model (1984 drugs out of a total of ~10 K available in DrugBank). The DrugCentral database (open database of drugs, June 2018) was used to identify drugs designed for primary use against cancer^47,48^. RepoDB (open database of repositioned drugs, Nov 2017) was used to identify drugs that have been successfully repositioned for anti-cancer purposes (secondary or tertiary use)^49^. For our machine-learning approach drugs designed and tested specifically for anticancer treatment (n = 199) were denoted as the ‘positive’ class and drugs with no known association with cancer were used as the ‘negative’ class (n = 1692). Drugs that have been repositioned for secondary/tertiary use in cancer have been excluded from the model. Drug compounds extracted from different databases were matched using InChI keys.

Drug-gene encoded protein interaction data were extracted from the STITCH database (open database of chemical-gene interactions, Nov 2017) and once more drug compounds were matched using InChI keys^50^. A significance score for individual drug-protein interactions was extracted from the STITCH database. Different levels of interaction significance as defined by threshold were considered as part of the computational strategy. Compounds from FooDB (open database of foods and food compounds, Jun 2018) for which InChI identifier was available were matched to STITCH in the same way as drugs to generate the scored list of compound-gene interactions^51^. The interactions were filtered according to the score threshold identical to the one used for the drugs in the model (the actual value is model-dependent). T3DB was used to highlight toxic and potentially toxic food compounds (matching performed using InChI keys)^52^.

### Compilation of human proteome network datasets

A human genome network of 20,256 proteins was compiled using data extracted from STRING^53^, UniProt^54^, COSMIC^55^, and NCBI Gene^56^ public databases. Due to the heterogeneity in gene/protein nomenclature in these databases, we used a sequence-based matching approach based on protein amino acid sequence alignment to establish the correspondence between proteins across databases. The amino acid sequences of 15911 proteins out of 20,256 were precisely matched between databases. The remaining sequences were then checked to determine if any were subsets of a larger amino acid sequence in any of the above databases. This permitted further alignment of 1532 protein sequences. Finally, the remaining proteins were aligned using ‘fuzzy’ matching (allowing up to 5% amino acid sequence mismatch) generating an additional 1686 proteins. Non-matched amino acid sequences (1,127) with their corresponding database identifiers were incorporated into the unified database. This resulted in 20,256 unique gene-encoded proteins and their identifiers/names/synonyms from different databases (including Ensembl ID, HGNC), where available.

Protein-protein interactions were imported from STRING resulting in ~ 11 million connections with the confidence scores in the range 0–999. Additionally, BioPlex, an open database of experimentally established protein-protein interactions, was mapped onto our gene list using gene id, Uniprot ID and gene name^57^. ~ 100 K connections for 10859 genes were added to the interactome network from BioPlex in addition to the ones imported from STRING.

Our observation showed full matching between Ensembl IDs from STRING and STITCH databases, providing a reliable link between chemical-protein and protein-protein interaction networks. Thus it was decided to use these two databases as a core model and reference for matching for other databases. Scored protein-protein interactions were imported from STRING into the propagation model with the score threshold used to filter out “unreliable” ones (adjustable parameter in the model).

### Unsupervised learning on graphs using random walks

The resulting interactome network was represented as a graph where nodes are gene-encoded proteins and the links between them correspond to biological interactivity. The graph makes no assumption regarding the direction of interaction between proteins (referred to as “undirected” graph). The link weights were dichotomized with various thresholds. The optimum threshold value was derived using a “nested” cross-validation strategy.

All proteins interacting with a given drug/bioactive molecule were assigned a value of 1.0 and all others were assigned the value of 0.0. This resulted in a sparse protein profile interacting with a given molecule (on average 20–30 targets per molecule). However on the understanding that these proteins act as part of the wider protein-protein network rather than in isolation, the unsupervised learning on graph algorithm (namely, a random walk with restarts) was applied to “learn” latent network-wide effects of a specific molecule. This network diffusion transforms a short list of proteins targeted by a given molecule/drug into a genome-wide profile of gene scores based on their network proximity to target candidates^24^.

From a computational perspective, we represent targeted proteins as “entry points” for a random walk which is defined as a path consisting of a succession of random steps within the interactome network. Before the iteration starts the probability of the walker to be in any of the ‘entry’ points is set to 1.0 divided by the number of ‘entry’ points, forming the starting sparse probability distribution vector, **p**_0_. The probability of transition from node ***a*** to a connected node ***b*** is given by 1.0 divided by the number of outgoing connections from node ***a***. These transition probabilities for the whole interactome form a scaled adjacency matrix, *W*. The probability of the walker to restart from its ‘entry’ point is given by the parameter “*c”*. This parameter denotes how far the influence of a given molecule spreads within the network with *c* = 1.0 meaning no propagation beyond ‘entry’ points, while *c* close to 0.0 would result in potential propagation to the furthest connected node(s), resulting in a “smoother” genome-wide profile. For each subsequent step of the algorithm the new distribution of the probabilities of finding the walker in any of the nodes **p**_i_ is given by Eq. 1:

1 $${{\bf{p}}}_{{\rm{i}}}={{\bf{p}}}_{{\rm{i}}-1}\ast W\ast (1.0-c)+c\ast {{\bf{p}}}_{0},$$

where **p**_i-1_ is the probability distribution from the previous iteration. The algorithm assumes convergence when |**p**_i_-**p**_i-1_| is less than a set tolerance value and the obtained probability distribution **p**_i_ (also referred to as “smoothed” genome-wide profile for a given molecule/drug) is returned for use in downstream supervised machine learning steps of the strategy^58^.

### Supervised machine-learning using propagated network profiles

Supervised-machine learning strategies based on Support Vector Machine (SVM)^29^ and Maximum Margin Criterion (“MMC”)^28^ were optimized to identify anti-cancer therapeutics based on their influence on diffused interactome profiles. The parameters for linear (“c”) and radial kernels (“c”, gamma) were optimized during SVM training. Both ‘positive’ and ‘negative’ classes of drugs formed the set used for model training. The best performing strategy (including type of interactome, parameter thresholds and settings for random walks on graphs, and supervised modeling methodology) was defined according to the F-score (balancing sensitivity and specificity) by a nested cross-validation strategy (see below). Due to the high class imbalance (~1:9 anti-cancer vs non-anticancer drugs), F-score was used as the main measuring criterion for the performance of the classifier. Stratified K-fold and “balanced” weights were used to compensate for class imbalance. The full list of parameter combinations tried with corresponding statistics is provided in SI Dataset S1. We also trained 2 convolutional neural network classifiers^59^ and 2 regularized LASSO/Elastic Net classifiers^60^ to see whether there is any improvement in classification performance for the best performing type of interactome and settings for random walk on graphs (see SI Appendix M1 for methodological details).

### Overall workflow for drug and active food molecules repurposing

Here, we assume that drugs/molecules acting on common protein networks (responsible for a variety of metabolic and signaling processes) should therefore exert similar downstream disease modifying effects. In order to validate this assumption and to predict unique anti-cancer compounds which could potentially be used/repositioned for cancer treatment we have tailored a bespoke machine learning strategy as outlined below:

1. The proteins interacting with molecular compounds (either existing drugs or bioactive compounds within foods) were mapped onto interactome;
2. The network-wide diffused effect of a given molecule was derived using a grid of different settings: the type of interactome network (BioPlex or STITCH), varying connection thresholds for the links between proteins (STRING, STITCH and BioPlex interactomes), and varying values of the “*c”* parameter in the random walk propagation algorithm);
3. A supervised-machine learning strategy based on SVM^29^, MMC and CNN algorithms^28^ was optimized to identify anti-cancer therapeutics based on their influence on diffused interactome networks.
4. Molecular anti-cancer “likeness” was calculated as the probability outcome of the best performing ML strategy (F-score ≥ 0.84, achieved by the 700 best performing models). These anti-cancer probability estimates were used to create a summary table of potential candidates for anti-cancer repurposing (SI Dataset S2).
5. Once validated on anti-cancer therapeutics, food compounds were processed in exactly the same way as the drugs used to train the models and then the best models obtained in the previous step were used to generate probabilistic predictions for the anti-cancer “likeness” of these food compounds (SI Dataset S3).
6. The list of the food compounds with the highest probability of exhibiting anti-cancer properties has been compiled and manually curated to exclude toxic compounds and compounds shown to promote cancer (the model is effective at highlighting both anti-cancer compounds and cancer-promoting compounds as they often share underlying biological mechanisms and interactions). Furthermore, compounds associated with normal metabolism of cells, *e.g*. dCTP belonging to the superclass of nucleosides, nucleotides, and analogues and directly involved in DNA synthesis were also removed from the final curated list. The compound-food associations were retrieved from the FooDB database. The curated results are provided as SI Appendix Tables 1&2.

### Nested Cross-Validation strategy

A 10-fold nested cross-validation strategy was employed to assess the predictive capacity of each method and model generated. Each test and training set split was stratified to keep equal proportions of ‘positive’ (anti-cancer therapeutics) and ‘negative’ (non anti-cancer therapeutics) classes in each split. For linear and radial SVM classifiers 5-fold inner cross-validation was used to optimize C and gamma parameters. Average per class classification accuracy and F-score metrics were used for the assessment of model predictive capacity due to class imbalance (~1:9 for ‘positive’:’negative’ classes). Logistic regression was employed for MMC as well as linear and radial SVMs to provide classification probability estimates. For each fold the anti-cancer “likeness” of a given molecule (based on its influence on interactome networks) in the test set was predicted. Averaged F-scores from 10-fold outer cross-validation was used to select the best ML strategy among all combinations of pre-processing, unsupervised and supervised model parameters (drug-gene connection confidence thresholds: 0, 100, 200, 325, 400, 500, 600, 700; gene-gene connection confidence thresholds: 400, 600, 700, 800, 850 or present in BioPlex; Random walk with restarts “*c*”: 0.0001, 0.001, 0.002, 0.004, 0.01, 0.015, 0.02, 0.03, 0.035, 0.04, 0.05, 0.076, 0.1, 0.2; preprocessing with log-transform: yes/no). The models were re-trained using the entire set of ‘positive’ and ‘negative’ classes (and the averaged best C and gamma, where applicable) prior to using them to predict anti-cancer “likeness” of the food compounds and the drugs which were not a part of the model building set. All tested parameterization sets and training statistics are provided in the SI Dataset S1.

### Pathway analytics

Pathway analytics was performed using gene set enrichment analysis via Python GSEAPY package^61^. Propagated gene/protein perturbation values were supplied as the input data for “*prerank”* module. Reactome_2016 and KEGG_2016 gene sets were used by default. Scored pathways were sorted by the normalized enrichment score reported by the script. Top 10 pathways for each gene collection and each CBM were reported in SI Dataset S3.

## Supplementary information

Supplementary information appendix

Dataset 1

Dataset 2

Dataset 3

Dataset 4

Dataset 5

## Data Availability

In order to allow for validation of methodology and results, we compiled a set of data processing python scripts, DRUGS project C++ code run in DreamLab App and the pre-processed input data (incl. gene connections and compound selections) in a form of an integral “container” for easy running on the individual PC (downloadable from https://bitbucket.org/iAnalytica/drugs_container_public/src/master/). The current container has the drug selections for anti-cancer compound identification, however, it can easily be supplemented with the other drug selections in order to re-use it to target other diseases and medical conditions. For more details see readme.md in the container. It has to be noted that the current container is focusing on full propagated profiles analysis presented in this paper and does not currently support combinatorial analysis or gene tailoring which are to be published later when ready within the scope of the DRUGS project.
