# Supplementary material for: HyperFoods: Machine intelligent mapping of cancer-beating molecules in foods
Source: Sci Rep. 2019 Jul 3;9:9237. doi: 10.1038/s41598-019-45349-y (PMC6610092; doi:10.1038/s41598-019-45349-y)
Supplement: Supplementary file 1 — Supplementary information appendix [file 41598_2019_45349_MOESM1_ESM.pdf]

Supplementary Information for

HyperFoods: Machine intelligent mapping of cancer-beating molecules in foods

Kirill Veselkov[a,1], Guadalupe Gonzalez Pigorini[a,c], Shahad Aljifri[a], Dieter Galea[a], Reza Mirnezami[a], Jozef Youssef[b], Michael Bronstein[c] and Ivan Laponogov[a]

[a]Department of Surgery and Cancer, Faculty of Medicine, Imperial College London, London SW7 2AZ, UK; [b]Kitchen Theory, UK; [c]Department of Computing, Faculty of Engineering, Imperial College London, London SW7 2AZ, UK.

Kirill Veselkov

Email: kirill.veselkov04@imperial.ac.uk

This PDF file includes:

Supplementary methods (M1)
Tables S1 to S2
Captions for datasets S1 to S5
References for SI reference citations

Supplementary methods (M1): Justification for the use of linear SVM and MMC

We also trained 2 neural networks and regularized LASSO/Elastic Net classifiers to see whether there is any improvement in classification performance for the best performing type of interactome and settings for random walk on graphs. The first NN-1 classifier had a fully-connected layer with a 2-dimensional output and softmax activation function to output probabilities of belonging to anticancer and non-anticancer classes. The second NN-2 classifier comprised a linear layer (with an output dimensionality of number of molecules - 1) and a fully-connected layer (with a 2-dimensional output) with softmax activation function. Both classifiers were trained using Momentum optimizer and l2 regularization. We used weighted cross-entropy as the cost function. Model performance was evaluated using 10-fold cross-validation. In the cross-validations, the training data was further split into training and validation set (10%), using the validation set for early stopping: training was stopped when either (i) the maximum number of epochs was reached (20K) or (ii) the validation loss continuously increased in a window of 5 evaluation steps (with evaluations every 50 epochs). For each fold, the model was saved when the validation loss was lowest and used for prediction on the test set. Cross-

validation experiments were done to find the optimal learning rate and l2 regularization hyper-parameter. Optimal values of learning rate and l2 regularization parameters were 10 and 1e-4 for the first classifier, and 1e-2 and 1 for the second classifier. Finally, regularized LASSO and Elastic Net classifiers were trained using stochastic gradient decent. The model parameters (alpha for LASSO and alpha/l1 for Elastic Net) were optimized using 10 fold nested cross validation. Final results (F-score) in 1:1 comparison were as follows:

1) LinearSVM: 84.7%
2) RadialSVM: 84.0%
3) LASSO: 82.7%
4) NN model 2: 81.3%
5) NN model 1: 80.1%
6) LASSO_logreg: 77.5%
7) Elastic Net: 72.9%
8) Elastic Net_logreg: 70.0%

Table S1. Cancer beating molecules in different foods

Common Name	Scientific Name	Number of CBMs	CBM Names
Tea	Camellia sinensis	17	1,2,4-Trihydroxybenzene; 6-Keto-28-homobrassinolide; Apigenin; Brassinolide; Epigallocatechin 3-gallate; Gallic acid; Gallocatechin 3-gallate; Lupeol; Phloroglucinol; Procyanidin B2; Procyanidin B3; Prodelphinidin B4; Quercetin; Theaflavin; Tricetin; α-Terpineol; ent-Epigallocatechin 3-gallate; ent-Gallocatechin 3-gallate
Carrot, Wild carrot	Daucus carota	12	Aesculetin; Apigenin; Carvone; Diosgenin; Ferulic acid 4-glucoside; Lupeol; Myristicin; Psoralen; Quercetin; Xanthotoxin; α-Terpineol; β-Elemene
Common grape	Vitis vinifera	12	Anthocyanidins; Betulinic acid; Epigallocatechin 3-gallate; Gallic acid; Gallocatechin 3-gallate; Lupeol; Procyanidin B1; Procyanidin B2; Procyanidin B3; Quercetin; α-Terpineol; gamma-Tocotrienol
Dill	Anethum graveolens	12	(R)-Carvone; (S)-Carvone; Aesculetin; Apigenin; Apiole; Carvone; Myristicin; Quercetin; Umbelliprenin; Xanthotoxin; α-Terpineol; β-Elemene
Wild celery	Apium graveolens	12	8-p-Menthene-1,2-diol; Angelicin; Apigenin; Apiole; Carvone; Myristicin; Psoralen; Quercetin; Verbenol; Xanthotoxin; α-Terpineol; β-Elemene
Sweet bay	Laurus nobilis	11	(S)-α-Terpineol; Artecanin; Carvone; Cuminyl alcohol; Procyanidin B2; Quercetin; Reynosin; Ridentin B; Santamarin; α-Terpineol; β-Elemene
Caraway	Carum carvi	10	(R)-Carvone; (S)-Carvone; 8-p-Menthene-1,2-diol; Carvone; Myristicin; Quercetin; Xanthotoxin; α-Terpineol; β-Elemene; gamma-Tocotrienol
Sweet orange	Citrus sinensis	10	Brassinolide; Carvone; Didymin; Luteolin 7-rhamnosylglucoside; Obacunone; Quercetagetin; Quercetin; Xylan; α-Terpineol; β-Elemene
Coriander	Coriandrum sativum	9	Angelicin; Apigenin; Carvone; Myristicin; Psoralen; Quercetin; Umbelliprenin; Xanthotoxin; α-Terpineol
Guava	Psidium guajava	9	(15Z)-Lycopene; Corosolic acid; Gallic acid; Lupeol; Procyanidin B1; Procyanidin B2; Procyanidin B3; Quercetin; α-Terpineol
Rosemary	Rosmarinus officinalis	9	Apigenin; Betulinic acid; Carnosic acid; Carvone; Lupeol; Verbenol; α-Terpineol; β-Elemene; gamma-Tocotrienol

Lovage	Levisticum officinale	8	(Z)-3-Butylidene-1(3H)-isobenzofuranone; Apiole; Carvone; Myristicin; Psoralen; Xanthotoxin; α-Terpineol; β-Elemene
Soy bean	Glycine max	8	6-Hydroxymethylpterin; Fisetin; Gallic acid; Genistein; Glyceollin I; Legumelin; Lupeol; Quercetin
Anise	Pimpinella anisum	8	(S)-Carvone; Apigenin; Carvone; Myristicin; Quercetin; Xanthotoxin; α-Terpineol; β-Elemene
Broad bean	Vicia faba	8	3,4,7-Trihydroxyflavone; 4-Chloro-1H-indole-3-acetic acid; 7,4-Dihydroxyflavone; Brassinolide; Genistein; Procyanidin B1; Procyanidin B3; Prodelphinidin B4
Cabbage, White cabbage	Brassica oleracea	8	1H-Indole-3-methanol; 4-Methoxyglucobrassicin; Brassinin; Carvone; Erucin; Indole-β-carboxylic acid; Quercetin; α-Terpineol
Common sage	Salvia officinalis	8	6-Oxocamphor; Apigenin; Carnosic acid; Epiursolic acid; Gallic acid; Lupeol; Manool; α-Terpineol
Fennel	Foeniculum vulgare	8	Apiole; Carvone; Myristicin; Psoralen; Quercetin; Xanthotoxin; α-Terpineol; gamma-Tocotrienol
Olive	Olea europaea	8	Acteoside; Aesculetin; Apigenin; Betulinic acid; Corosolic acid; Gallic acid; Hydroxytyrosol; Quercetin
Parsley	Petroselinum crispum	8	Apigenin; Apiole; Myristicin; Psoralen; Quercetin; Xanthotoxin; α-Terpineol; β-Elemene
Pomegranate	Punica granatum	8	Apigenin; Betulinic acid; Epiestradiol; Epigallocatechin 3-gallate; Gallic acid; Procyanidin B1; Procyanidin B2; Quercetin
Sweet basil	Ocimum basilicum	8	Aesculetin; Apigenin; Betulinic acid; Carvone; Quercetin; α-Santalene; α-Terpineol; β-Elemene
Cloves	Syzygium aromaticum	7	Carvone; Gallic acid; Hydroxytyrosol; Palustrol; Procyanidin; Quercetin; α-Terpineol
Common oregano	Origanum vulgare	7	Apigenin; Carvone; Gallic acid; Quercetin; Tetramethylquercetin; α-Terpineol; β-Elemene
Highbush blueberry	Vaccinium corymbosum	7	Aesculetin; Ferulic acid 4-glucoside; Gallic acid; Myristicin; Phloroglucinol; Quercetin; α-Terpineol
Lemon	Citrus limon	7	Carvone; Luteolin 7-rhamnosylglucoside; Obacunone; Quercetin; Umbelliprenin; α-Terpineol; β-Elemene
American cranberry	Vaccinium macrocarpon	6	Caffeic acid 3-glucoside; Myricetin 3-digalactoside; Procyanidin B2; Quercetin; α-Terpineol; gamma-Tocotrienol
Apple	Malus domestica	6	Apigenin; Gallic acid; Procyanidin B1; Procyanidin B2; Progesterone; Quercetin
Bilberry	Vaccinium myrtillus	6	Gallic acid; Procyanidin B1; Procyanidin B2; Procyanidin B3; Quercetin; α-Terpineol
Corn	Zea mays	6	Gallic acid; Genistein; Quercetin; Xanthotoxin; α-Terpineol; gamma-Tocotrienol
Fenugreek	Trigonella foenum-graecum	6	3,4,7-Trihydroxyflavone; 7,4-Dihydroxyflavone; Apigenin; Diosgenin; Genistein; Quercetin
Fig	Ficus carica	6	Angelicin; Lupeol; Psoralen; Quercetin; Xanthotoxin; α-Terpineol
Ginkgo nuts	Ginkgo biloba	6	Apigenin; Ginkgetin; Kaempferol 3-rhamnoside; Procyanidin; Quercetin; Tricetin
Pepper (Spice)	Piper nigrum	6	Carvone; Myristicin; Quercetin; α-Santalene; α-Terpineol; β-Elemene
Potato	Solanum tuberosum	6	2-Propanethiol; Eicosan-1-ol; Gallic acid; Progesterone; Quercetin; β-Elemene
Spearmint	Mentha spicata	6	(R)-Carvone; Apigenin; Carvone; Verbenol; α-Terpineol; β-Elemene
Sunflower	Helianthus annuus	6	Brassinolide; Cuminyl alcohol; Dioxindole-3-acetic acid; Lupeol; Quercetin; α-Terpineol
Tarragon	Artemisia dracunculus	6	Aesculetin; Apigenin; Carvone; Gallic acid; Phloroglucinol; Quercetin
Black elderberry	Sambucus nigra	5	Betulinic acid; Eicosan-1-ol; Lupeol; Quercetin; α-Terpineol
Chicory	Cichorium intybus	5	Aesculetin; Apigenin; Betulinic acid; Lupeol; Quercetin
Common hazelnut	Corylus avellana	5	Kaempferol 3-rhamnoside; Procyanidin B1; Procyanidin B3; Quercetin; Xylan
Common pea	Pisum sativum	5	4-Chloro-1H-indole-3-acetic acid; Genistein; Indole-β-carboxylic acid; Lupeol; Progesterone
Common thyme	Thymus vulgaris	5	Apigenin; Carvone; Cuminyl alcohol; Gallic acid; α-Terpineol

Garden tomato (var.)	Lycopersicon esculentum	5	Ferulic acid 4-glucoside; Lupeol; Progesterone; Prolycopene; Quercetin
Mandarin orange (Clementine, Tangerine)	Citrus reticulata	5	Carvone; Obacunone; Tetramethylquercetin; α-Terpineol; β-Elemene
Parsnip	Pastinaca sativa	5	Angelicin; Myristicin; Psoralen; Quercetin; Xanthotoxin
Peanut	Arachis hypogaea	5	Aesculetin; Dihydroxystearic acid; Procyanidin B2; Procyanidin B3; Quercetin
Soft-necked garlic	Allium sativum	5	Ajoene; Apigenin; Di-2-propenyl sulfide; Phloroglucinol; Quercetin
Allspice	Pimenta dioica	4	Gallic acid; α-Santalene; α-Terpineol; β-Elemene
Barley	Hordeum vulgare	4	Aesculetin; Indole-β-carboxylic acid; Procyanidin B3; gamma-Tocotrienol
Ceylon cinnamon	Cinnamomum verum	4	Procyanidin B1; Procyanidin B2; α-Terpineol; β-Elemene
Cocoa bean	Theobroma cacao	4	Aesculetin; Procyanidin B1; Procyanidin B2; Quercetin
Common bean	Phaseolus vulgaris	4	Aesculetin; Apigenin; Brassinolide; Genistein
Common buckwheat	Fagopyrum esculentum	4	Brassinolide; Dodecadienoic acid; Procyanidin B2; Quercetin
Common walnut	Juglans regia	4	Betulinic acid; Gallic acid; Plumbagin; Quercetin
Dandelion	Taraxacum officinale	4	Aesculetin; Dodecadienoic acid; Luteolin 7-rhamnosylglucoside; Quercetin
Garden onion	Allium cepa	4	Di-2-propenyl sulfide; Phloroglucinol; Progesterone; Quercetin
Ginger	Zingiber officinale	4	Quercetin; [8]-Shogaol; α-Terpineol; β-Elemene
Pepper (C. frutescens)	Capsicum frutescens	4	Carvone; Dihydrocapsaicin; Quercetin; α-Terpineol
Peppermint	Mentha x	4	(R)-Carvone; Carvone; Cuminyl alcohol; α-Terpineol
Pigeon pea	Cajanus cajan	4	Cajanin; Cajanol; Genistein; Lupeol
Sour cherry	Prunus cerasus	4	Apigenin; Gallic acid; Genistein; Quercetin
Star anise	Illicium verum	4	Carvone; α-Santalene; α-Terpineol; β-Elemene
Turmeric	Curcuma longa	4	Cuminyl alcohol; Demethoxycurcumin; Quercetin; α-Terpineol
Alfalfa	Medicago sativa	3	3,4,7-Trihydroxyflavone; 7,4-Dihydroxyflavone; Genistein
Apricot	Prunus armeniaca	3	Epiestradiol; Quercetin; α-Terpineol
Avocado	Persea americana	3	Hentriacosane; Procyanidin C; Quercetin
Black walnut	Juglans nigra	3	Gallic acid; Plumbagin; Quercetin
Blackcurrant	Ribes nigrum	3	Prodelphinidin B4; Quercetin; α-Terpineol
Capers	Capparis spinosa	3	4-Methoxyglucobrassicin; Quercetin; Rutinose
Cardamom	Elettaria cardamomum	3	(S)-α-Terpinyl acetate; Carvone; α-Terpineol
Carob	Ceratonia siliqua	3	Gallic acid; Phloroglucinol; Quercetin
Chinese cinnamon	Cinnamomum aromaticum	3	Procyanidin B1; Procyanidin B2; α-Terpineol
Cinnamon	Cinnamomum spp.	3	(S)-α-Terpineol; Procyanidin B1; Procyanidin B2
Common persimmon	Diospyros virginiana	3	Betulinic acid; Lupeol; Quercetin
Cornmint	Mentha arvensis	3	(R)-Carvone; (S)-Carvone; Carvone
Cucumber	Cucumis sativus	3	Cucurbitacin I; Indole-β-carboxylic acid; Lupeol
Date	Phoenix dactylifera	3	Apigenin; Lupeol; Quercetin
Evening primrose	Oenothera biennis	3	Gallic acid; Lupeol; Quercetin
Flaxseed	Linum usitatissimum	3	Apigenin; Eicosan-1-ol; Secoisolariciresinol 9,9-diglucoside
Garden rhubarb	Rheum rhabarbarum	3	Emodin; Gallic acid; Isorhapontigenin

German camomile	Matricaria recutita	3	Apigenin; Luteolin 7-rhamnosylglucoside; Quercetin
Green bell pepper, Orange bell pepper, Pepper (C. annuum), Red bell pepper, Yellow bell pepper	Capsicum annuum	3	Dihydrocapsaicin; Lupeol; α-Terpineol
Horseradish	Armoracia rusticana	3	Aesculetin; Di-2-propenyl sulfide; Quercetin
Hyssop	Hyssopus officinalis	3	Verbenol; α-Terpineol; β-Elemene
Japanese persimmon	Diospyros kaki	3	Betulinic acid; Lupeol; Plumbagin
Linden	Tilia sp.	3	Kaempferol 3-rhamnoside; Quercetin; α-Terpineol
Loquat	Eriobotrya japonica	3	Corosolic acid; Procyanidin B2; Quercetin
Mango	Mangifera indica	3	Gallic acid; Mangiferol; Quercetin
Mung bean	Vigna radiata	3	Genistein; Progesterone; Quercetin
Nutmeg	Myristica fragrans	3	Myristicin; Quercetin; α-Terpineol
Orange mint	Mentha aquatica	3	Apigenin; Carvone; β-Elemene
Peach	Prunus persica	3	Anthocyanidins; Kaempferol 3-rhamnoside; Quercetin
Pot marjoram	Origanum onites	3	Carvone; Cuminyl alcohol; α-Terpineol
Radish	Raphanus sativus	3	Brassinolide; Di-2-propenyl sulfide; Gibberellin A116
Rice	Oryza sativa	3	Progesterone; Quercetin; gamma-Tocotrienol
Sea-buckthornberry	Hippophae rhamnoides	3	Gallic acid; Gallocatechin 3-gallate; Quercetin
Star fruit	Averrhoa carambola	3	Epigallocatechin 3-gallate; Procyanidin B1; Procyanidin B2
Winter savory	Satureja montana	3	Apigenin; Carvone; α-Terpineol
Cumin	Cuminum cyminum	2	α-Terpineol; β-Elemene
Adzuki bean	Phaseolus angularis	2	Genistein; Progesterone
Arabica coffee	Coffea arabica	2	Cafestol; Xylan
Asparagus	Asparagus officinalis	2	Diosgenin; Quercetin
Bitter gourd	Momordica charantia	2	Acteoside; Diosgenin
Burdock	Arctium lappa	2	Lupeol; β-Elemene
Chinese cabbage	Brassica rapa	2	4-Methoxyglucobrassicin; Brassinin
Coconut	Cocos nucifera	2	α-Terpineol; gamma-Tocotrienol
Common beet	B vulgaris	2	Indole-β-carboxylic acid; Quercetin
Common verbena	Verbena officinalis	2	Lupeol; Verbenalin
Common wheat	Triticum aestivum	2	Apigenin; Quercetin
Eggplant	Solanum melongena	2	Solamargine; Solasonine
European chestnut	Castanea sativa	2	Gallic acid; Quercetin
Gram bean	Vigna mungo	2	Diethylstilbesterol; Genistein
Hyacinth bean	Lablab purpureus	2	Brassinolide; Genistein
Java plum	Syzygium cumini	2	Betulinic acid; Gallic acid
Lemon balm	Melissa officinalis	2	α-Terpineol; β-Elemene
Lemon grass	Cymbopogon citratus	2	Quercetin; α-Terpineol
Lentils	Lens culinaris	2	4-Chloro-1H-indole-3-acetic acid; Genistein
Lime	Citrus aurantiifolia	2	α-Terpineol; β-Elemene
Lingonberry	Vaccinium vitis-idaea	2	Procyanidin B1; Procyanidin B3

Malus (Crab apple)	Malus spp.	2	Procyanidin B1; Procyanidin B2
Mentha (Mint)	Mentha spp.	2	(S)-α-Terpineol; β-Elemene
Mugwort	Artemisia vulgaris	2	Aesculetin; α-Terpineol
Oat	Avena sativa	2	Quercetin; gamma-Tocotrienol
Pineapple	Ananas comosus	2	Ergosterol peroxide; α-Terpineol
Red raspberry	Rubus idaeus	2	Gallic acid; Quercetin
Rocket salad (ssp.)	Eruca sativa	2	Carvone; Cuminyl alcohol
Rubus (Blackberry, Raspberry)	Rubus spp.	2	Procyanidin B1; Procyanidin B3
Sesame	Sesamum indicum	2	Acteoside; Sesamolin
Sorrel	Rumex acetosa	2	Emodin; Quercetin
Spinach	Spinacia oleracea	2	Crustecdysone; Quercetin
Swede	Brassica napus	2	4-Methoxyglucobrassicin; Brassinolide
Sweet marjoram	Origanum majorana	2	Carvone; α-Terpineol
Tamarind	Tamarindus indica	2	α-Terpineol; β-Elemene
White lupine	Lupinus albus	2	Genistein; Lupeol
Wild leek	Allium ampeloprasum	2	Aesculetin; Di-2-propenyl sulfide
Abiyuch	Crateva religiosa	1	Betulinic acid
Almond	Prunus dulcis	1	Quercetin
Angelica	Angelica keiskei	1	Xanthotoxin
Arrowroot	Maranta arundinacea	1	Quercetin
Black-eyed pea	Vigna unguiculata	1	Genistein
Borage	Borago officinalis	1	Quercetin
Breadfruit	Artocarpus altilis	1	Quercetin
Cashew nut	Anacardium occidentale	1	Gallic acid
Cherimoya	Annona cherimola	1	Liriodenine
Chickpea	Cicer arietinum	1	Genistein
Chinese mustard	Brassica juncea	1	4-Methoxyglucobrassicin
Chives	Allium schoenoprasum	1	Quercetin
Coffee	Coffea spp.	1	Cafestol
Cottonseed	Gossypium sp	1	Quercetin
Custard apple	Annona reticulata	1	Liriodenine
Dock	Rumex spp.	1	Emodin
Endive	Cichorium endivia	1	Quercetin
European plum	Prunus domestica	1	Quercetin
French plantain	Musa x	1	Quercetin
Garden cress	Lepidium sativum	1	Di-2-propenyl sulfide
Giant butterbur	Petasites japonicus	1	α-Santalene
Grass pea	Lathyrus sativus	1	4-Chloro-1H-indole-3-acetic acid
Horseradish tree	Moringa oleifera	1	Quercetin
Jackfruit	Artocarpus heterophyllus	1	Betulinic acid
Japanese chestnut	Castanea crenata	1	Brassinolide

Kiwi	Actinidia chinensis	1	Quercetin
Lambsquarters	Chenopodium album	1	Xanthotoxin
Lettuce	Lactuca sativa	1	Quercetin
Lichee	Litchi chinensis	1	gamma-Tocotrienol
Lima bean	Phaseolus lunatus	1	Genistein
Malabar spinach	Basella alba	1	Quercetin
Mulberry	Morus sp.	1	Moracin P
Muskmelon	Cucumis melo	1	Lupeol
Nance	Byrsonima crassifolia	1	Betulinic acid
Okra	Abelmoschus esculentus	1	Quercetin
Opium poppy	Papaver somniferum	1	Aesculetin
Passion fruit	Passiflora edulis	1	Prolycopene
Pear	Pyrus communis	1	Quercetin
Persimmon	Diospyros spp.	1	Plumbagin
Prunus (Cherry, Plum)	Prunus spp	1	Genistein
Roman camomile	Chamaemelum nobile	1	Apigenin
Rose hip	Rosa spp	1	Quercetin
Roselle	Hibiscus sabdariffa	1	α-Terpineol
Rye	Secale cereale	1	gamma-Tocotrienol
Sacred lotus	Nelumbo nucifera	1	Quercetin
Saffron	Crocus sativus	1	Quercetin
Sapodilla	Manilkara zapota	1	Gallic acid
Scarlet bean	Phaseolus coccineus	1	Genistein
Shea tree	Vitellaria paradoxa	1	Lupeol
Soursop	Annona muricata	1	Procyanidin
Strawberry guava	Psidium cattleianum	1	Quercetin
Summer savory	Satureja hortensis	1	α-Terpineol
Sweet cherry	Prunus avium	1	Genistein
Sweet potato	Ipomoea batatas	1	Quercetin
Turnip	Brassica campestris	1	Brassinolide
Wakame	Undaria pinnatifida	1	Indole-β-carboxylic acid
Winged bean	Psophocarpus tetragonolobus	1	Betulinic acid
Yam	Dioscorea sp.	1	Diosgenin

Table S2. List of machine learning-predicted compounds in foods and their anti-cancer likeness (only compounds with anti-cancer likeness of over 90% were selected for literature review, compounds with little evidence in the literature or known toxicity/carcinogenicity were excluded later on).

Name/Class	AC likeness	Experimental Supporting evidence	Food types
Artecanin	1.00	Anti-proliferative actions: prevention of metastasis, inhibition of inflammatory responses, and induction of apoptosis with the effects on cell signalling pathways such as nuclear transcription factor-kappaB (NF-κB) and mitogen-activated protein kinases (MAPK) [1,2]	Sweet bay
6-Oxocamphor /topotecan/ hycampin	1.00	Anti-cancer activity by interfering with the action of topoisomerase enzymes which control cell division and replication [3,4];	Common sage
Lupeol	1.00	Chemo-preventative and hemotherapeutics effects by targeting key molecular pathways which involve NF-κB, cFLIP, FAS, KRAS, PI3K/AKT and WNT/β-catenin in a variety of cancer cells [5,6]	Black elderberry; Burdock; Carrot, Wild carrot; Chicory; Common grape; Common pea; Common persimmon; Common sage; Common verbena; Cucumber; Date; Evening primrose; Fig; Garden tomato (var.); Green bell pepper, Orange bell pepper, Pepper (C. annuum), Red bell pepper, Yellow bell pepper; Guava; Japanese persimmon; Muskmelon; Pigeon pea; Rosemary; Shea tree; Soy bean; Sunflower; Tea; White lupine
Procyanidin	1.00	Anti-inflammatory, anti-proliferative, and antitumor activities with numerous targets on proinflammatory mediators, regulators of cell survival and apoptosis, and angiogenic and metastatic mediators. Chemo-preventative and therapeutic activity demonstrated across a large array of cancer types [7].	Cloves; Ginkgo nuts; Soursop
Ajoene	0.99	Active at inhibiting the proliferation of tumor cells both *in vitro* and *in vivo via* assumed mechanism of activation of the mitochondrial-dependent caspase cascade [8,9].	Soft-necked garlic
Apiole	0.99	Induced cell-cycle arrest and apoptosis in human colorectal tumour cells *in vitro* and *in vivo* through activation of p53 signalling and caspase cascade [10]	Dill; Fennel; Lovage; Parsley; Wild celery
Cucurbitacin I	0.99	*In vitro* and *in vivo* anticancer activities ranging from anti-proliferation, cell cycle arrest to induction of apoptosisvia JAK/STAT, MAPK pathways, PARP cleavage, expression of active caspase-3 etc [11,12].	Cucumber
Didymin	0.99	*In vitro* and *in vivo* anti-cancer activity through Fas-mediated apoptotic pathway as well as inhibition of the proliferation and angiogenesis through a network of mediators (including MAPK-ERK pathway) regardless of the p53 status of cancer cells [13,14]	Sweet orange
Cajanol	0.99	Anti-proliferative, pro-apoptotic activities against cancer cells via ROS-mediated mitochondrial and ERα-dependent PI3K pathways [15,16]	Pigeon pea
Dioxindole-3-acetic acid	0.98	Pro-apoptotic activity against cancer cells via caspase signalling pathways [17,18]	Sunflower
Eicosan-1-ol	0.98	Anti-tumour properties of long-chain fatty	Black elderberry; Flaxseed; Potato

		alcohols includes the inhibition of angiogenesis and metastasis *in vivo* and *in vitro* by interacting with metalloproteinases activity and translocation of NG-kB to nucleolus [19]	
Cafestol	0.98	Anti-angiogenic, pro-apoptotic and anti-tumorigenic effects through a variety of processes such as PI3K/AKT and caspase signalling pathways [20,21]	Arabica coffee; Coffee
Manool	0.98	Induction of selective cytotoxicity in a variety of cancer cells [22]	Common sage
1H-Indole-3-methanol	0.98	Interact with multiple signalling pathways and target molecular networks controlling cell division, apoptosis, or angiogenesis deregulated in cancer cells [23,24]. These include AKT, NF-κB, BCL-2, MAPK and CDK signalling pathways.	Cabbage, White cabbage
Indole-beta-carboxylic acid	0.95		Barley; Cabbage, White cabbage; Common beet; Common pea; Cucumber; Wakame
4-Methoxy-glucobrassicin - converted to indole-3-methanol	0.97		Cabbage, White cabbage; Capers; Chinese cabbage; Chinese mustard; Swede
Legumelin/ Deguelin	0.98	Anti-tumor agent targeting apoptosis, cell cycle arrest and anti-angiogenesis for cancer chemoprevention and treatment via multiple mechanisms including PI3K/AKT signalling; anticancer and antimetastatic activity in part through downregulating GSK-3β/β-catenin signalling pathway and antiapoptotic survival proteins[25,26].	Soy bean
Ergosterol peroxide	0.98	Antitumor and anti-angiogenesis effects across different cancer types via β-catenin, p53, caspade and STAT3 signalling pathways and autophagy induction via JNK and ERK signalling with no toxicity observed in normal cells [27,28]	Pineapple
Sesamolin	0.98	Chemo protective and therapeutic effects against multiple cancer types via NF-kB, MAPK/ERK/JKN pathways [29]	Sesame
Verbenalin	0.98	Strong anti-inflammatory properties and induction of angiogenesis via a programmed PI3K/AKT/eNOS/VEGF signalling axis [30,31].	Common verbena
Phloroglucinol	0.98	Anti-metastatic, pro-apoptotic effects via IGF-1 signalling and Inhibition of KRAS and its downstream PI3K/AKT and RAF-1/ERK signaling pathways that regulate cancer cells [32,33]	Carob; Garden onion; Highbush blueberry; Soft-necked garlic; Tarragon; Tea
Tricetin	0.97	Anti-metastatic and pro-apoptotic effects against multiple cancer types *via* ROS-mediated JKN and AKT signalling pathways [34,35]	Ginkgo nuts; Tea
Caffeic acid	0.97	Anti-inflammatory and pro-apoptotic effects particularly against pro-oxidant-mediated oxidative DNA damage [36,37]	American cranberry
Ferulic acid	0.97	Pro-apoptotic effects, induction of autophagy and suppression of metastatic potential across different cancer types by scavenging free radicals, stimulating cytoprotective enzymes and inhibiting cytotoxic systems. Induction of angiogenesis via VEGF pathway [38,39]	Carrot, Wild carrot; Garden tomato (var.); Highbush blueberry
Verbenol	0.97	Strong anti-inflammatory and anti-proliferative activity mediated via NF-kB pathways and suppression of pro-inflammatory mRNA markers [40]	Hyssop; Rosemary; Spearmint; Wild celery
Obacunone	0.96	*In vivo* and *in vitro* anti-proliferative, pro-apoptotic and anti-inflammatory activity across multiple cancer types *via* MAPK signalling pathway with the downstream impact on COX-2/NF-kB/iNOS axis [41,42]	Lemon; Mandarin orange (Clementine, Tangerine); Sweet orange
Procyanidin B2	0.96	Promising lead compounds for cancer	American cranberry; Apple; Bilberry;

		prevention and treatments inhibiting the proliferation of various cancer cells in *in vitro* and *in vivo*. Anti-inflammatory, anti-proliferative, and antitumor activities with numerous targets on inflammatory mediators, regulators of cell survival and apoptosis, and angiogenesis.	Ceylon cinnamon; Chinese cinnamon; Cinnamon; Cocoa bean; Common buckwheat; Common grape; Guava; Loquat; Malus (Crab apple); Peanut; Pomegranate; Star fruit; Sweet bay; Tea
Procyanidin B3	0.96		Barley; Bilberry; Broad bean; Common grape; Common hazelnut; Guava; Lingonberry; Peanut; Rubus (Blackberry, Raspberry); Tea
Procyanidin C	0.96		Avocado
Procyanidin B1	0.89		Apple; Bilberry; Broad bean; Ceylon cinnamon; Chinese cinnamon; Cinnamon; Cocoa bean; Common grape; Common hazelnut; Guava; Lingonberry; Malus (Crab apple); Pomegranate; Rubus (Blackberry, Raspberry); Star fruit
Prodelphinidin B4	0.96	Anti-tumour effects via caspase and p53 signalling and anti-inflammatory properties via inhibition of COX-2 and iNOS via the downregulation of TAK1/NF-kB pathways (43, 44)	Blackcurrant; Broad bean; Tea
Solamargine	0.96	Anticancer activities via its effect on a variety of biological pathways including cell survival pathways, tumor suppressor pathways, caspase and death receptor pathways promote invasion/migration and multi drug resistance [43]	Eggplant
6-Keto-28-homobrassinolide	0.95	Growth inhibition of several human cancer cell lines without affecting the growth of normal cells mediated primary via pro-apoptotic effects (Bcl-2) [44,45]	Tea
Progesterone	0.95	Exogenous progesterone potently inhibit tumorigenesis and prevent the development of breast and ovarian cancers *in vivo* and *in vitro* potentially via p53 signalling and other pathways [46,47]In clinical trials	Adzuki bean; Apple; Common pea; Garden onion; Garden tomato (var.); Mung bean; Potato; Rice
Demethoxycurcumin	0.94	Inhibition of cell proliferation, migration and invasion across several cancer types by suppressing oxidate stress, caspase-dependent and NF-kB pathways [48,49]	Turmeric
Cajanin	0.94	Multiple ant-cancer effects including cell induction of apoptosis and cell cycle arrest via p53 signalling pathway; potential for anti-estrogenic therapy due to inhibition of ERalpha [50,51]	Pigeon pea
Betulinic acid	0.94	Exert potent anti-cancer effects *in vitro* and *in vivo* via a direct effect on mitochondria and induction of autophagy; anti-metastatic and anti-inflammatory properties [52,53]	Abiyuch; Black elderberry; Chicory; Common grape; Common persimmon; Common walnut; Jackfruit; Japanese persimmon; Java plum; Nance; Olive; Pomegranate; Rosemary; Sweet basil; Winged bean
Palustrol	0.94	Belong to a class of sesquiterpenoids which have shown to exert potent cytotoxic activity across multiple cell lines but require further *in vitro* and *in vivo* investigations [54].	Cloves
Carvone, (R)-Carvone, (S)-Carvone	0.94	Multiple anti cancer effects including inhibition of cancer cell invasion, induction of apoptosis via p53, p58, ERK and caspase signalling pathways, potentially immunomodulatory and anti-inflammatory effects [55,56].	Anise; Cabbage, White cabbage; Caraway; Cardamom; Carrot, Wild carrot; Cloves; Common oregano; Common thyme; Coriander; Cornmint; Dill; Fennel; Lemon; Lovage; Mandarin orange (Clementine, Tangerine); Orange mint; Pepper (C. frutescens); Pepper (Spice); Peppermint; Pot marjoram; Rocket salad (ssp.); Rosemary; Spearmint; Star anise; Sweet basil;

			Sweet bay; Sweet marjoram; Sweet orange; Tarragon; Wild celery; Winter savory; Dill
gamma-Tocotrienol	0.93	Targeting multiple pro-carcinogenic pathways inlcuding NF-κB, signal transducer and activator of transcription (STAT) 3, death receptors, apoptosis, Nrf2, HIF1, growth factor receptor kinases, and angiogenic pathways [57].	American cranberry; Barley; Caraway; Coconut; Common grape; Corn; Fennel; Lichee; Oat; Rice; Rosemary; Rye
(S)-α-Terpinyl acetate	0.92	Induction of apoptosis, cancer cellular growth and cell cycle regulation via ERK5, ILK signalling as well as endocytic pathways and p53 signalling [58]	Cardamom
Santamarin	0.92	Anti-inflammatory, and anticancer activities via oxidative stress and NF-kB and STAT3 signalling pathways [59]	Sweet bay
Diethylstilbesterol	0.92	Synthetic, nonsteroidal form of estrogen. Used in treatment of prostate and breast cancer and for hormone replacement therapy in postmenopausal women. Diethylstilbestrol was developed to supplement a woman's natural estrogen production. In 1971, the Food and Drug Administration (FDA) issued a Drug Bulletin advising physicians to stop prescribing DES to pregnant women because it was linked to a rare vaginal cancer in female offspring (DB00255- DrugBank,).	Gram bean
Reynosin	0.92	Anti-cancer activity potentially via caspase mediated signalling [60]	Sweet bay
Ridentin B	0.92	Belong to a class of sesquiterpene lactones which have demonstrated anti-cancer potential via interacting with cell NF-kB and MAPK signalling pathways [2].	Sweet bay
8-Shogaol	0.92	Inhibition of cell invasion via via blockade of NF-κB activation; pro-apoptotic effects v*ia* AKT and caspase signalling; effective against apoptosis resistance cancer stem cells [61,62]	Ginger
Diosgenin	0.92	Anti-tumour mechanisms via the modulation of multiple cell signalling pathways, which are associated with growth, differentiation, apoptosis and oncogenesis [63].	Asparagus; Bitter gourd; Carrot, Wild carrot; Fenugreek; Yam
Umbelliprenin	0.92	*In vivo* chemo-protective and antitumor effects: reduction of tumor size, angiogenesis, proliferation markers and anti-metastatic activity [64,65]	Coriander; Dill; Lemon
8-p-Menthene-1,2-diol	0.91	Potential antitumor activity by inducing autophagy and apoptosis; anti-metastatic activity [66,67]	Caraway; Wild celery
α-Terpineol	0.90	Potential Anticancer Agent which mainly acts through suppressing NF-κB signalling [68]	Allspice; American cranberry; Anise; Apricot; Bilberry; Black elderberry; Blackcurrant; Cabbage, White cabbage; Caraway; Cardamom; Carrot, Wild carrot; Ceylon cinnamon; Chinese cinnamon; Cloves; Coconut; Common grape; Common oregano; Common sage; Common thyme; Coriander; Corn; Cumin; Dill; Fennel; Fig; Ginger; Green bell pepper, Orange bell pepper, Pepper (C. annuum), Red bell pepper, Yellow bell pepper; Guava; Highbush blueberry; Hyssop; Lemon balm; Lemon grass; Lemon; Lime; Linden;

			Lovage; Mandarin orange (Clementine, Tangerine); Mugwort; Nutmeg; Parsley; Pepper (C. frutescens); Pepper (Spice); Peppermint; Pineapple; Pot marjoram; Roselle; Rosemary; Spearmint; Star anise; Summer savory; Sunflower; Sweet basil; Sweet bay; Sweet marjoram; Sweet orange; Tamarind; Tea; Turmeric; Wild celery; Winter savory

PhosphatidylInositol-3-Kinase (**PI3K**); Mitogen-Activated Protein Kinases (**MAPK**); Cellular FLICE (FADD-like IL-1β-converting enzyme)-inhibitory protein (**c-FLIP**); Protein kinase B **(AKT);** glycogen synthase kinase-3 beta (GSK-3β)/β-catenin; B-cell lymphoma 2 (Bcl-2); Wingless/Integrated (**WNT**); Cyclin-dependent kinases (**CDKs**); Janus kinases (JAKs), Signal Transducer and Activator of Transcription proteins (**STAT**s); Nuclear Factor-κB (**NF-kB**);, nuclear factor (erythroid-derived 2)-like 2 (**Nrf2**), hypoxia-inducible factor (**HIF**), extracellular signal-regulated kinases (**ERKs**); , integrin-linked kinase (**ILK**); mitogen-activated protein kinases (**MAPK**); c-Jun N-terminal kinase(**JNK**); inducible Nitric Oxide Synthase (**iNOS**); cyclooxygenase **(COX);** mechanistic target of rapamycin **(mTOR);** Vascular endothelial growth factor (**VEGF**); Transforming growth factor beta-activated kinases (**TAK**s); Reactive oxidative species (**ROS**); Poly (ADP-ribose) polymerase (**PARP**);

Additional dataset S1 (separate file): Model parameters and scores
List of all used parameter combinations and their corresponding F-scores and individual AC (anti-cancer, "positive" class) and non-AC ("other", "negative" class) fractions of correctly predicted labels.

Additional dataset S2 (separate file): Anti-cancer likeness drug prediction gene scores
Higher score means higher correlation between anti-cancer likeness of the drug and higher propagated gene value.

Additional dataset S3 (separate file): Anti-cancer likeness prediction for existing FDA approved drugs
Anticancer label columns reflect if the drug was marked as anti-cancer ("1") or not ("0") in DrugBank, repoDB or DrugCentral (as indication or off-label use). Column "Any" summarizes the labels. Anticancer likeness is the Platt/Logistic Regression probability of the compound being an anti-cancer drug and is an averaged prediction from the selected best 700 models with F-score >=0.84. Different models have different compound-gene connection confidence thresholds. Some compounds could not be analyzed by models with higher thresholds because none of the compound connections passed them. Model count indicates how many models could make the prediction for the compound in the list and reflects the reliability/strength of the prediction. The default decision threshold for anticancer likeness is 0.5. However, some compounds with the predicted anticancer likeness as low as of 0.09 were retrospectively found to be reported in the literature as potential anti-cancer candidates. Thus the list of compounds is reported up to the anticancer likeness of 0.09. Candidates with less than 90 models are excluded as unreliable.

Additional dataset S4 (separate file): Anti-cancer likeness prediction for the food compounds from FooDB
Anticancer likeness is the Platt/Logistic Regression probability of the compound being an anti-cancer drug and is an averaged prediction from the selected best 700 models with f-score >=0.84. Different models have different compound-gene connection confidence thresholds. Some compounds could not be analyzed by models with higher thresholds because none of the compound connections passed them. Model count indicates how many models could make the prediction for the compound in the list and reflects the reliability/strength of the prediction. Only compounds for which half of the models gave predictions were reported. Both cancerogenic and anti-cancer compounds could potentially be highlighted by this method. Some compounds were found to be present in toxin database as well (T3DB) (InChI Key matching, stereo isomers treated as the same molecule) and were supplemented with additional information about their mechanism of toxicity and carcinogenicity if available. Other fields cover compound classification based on FooDB, their description, top 10 pathways from Reactome and KEGG affected according to GSEA (Gene Set Enrichment Analysis), top 10 propagated genes, InChI keys and synonyms from FooDB. PubChem IDs are also provided for all compounds. The list of compounds is reported up to the anticancer likeness of 0.09 and candidates with less than 90 models are excluded as unreliable as in the case for anti-cancer drugs in SI Dataset S2.

Additional dataset S5 (separate file): Pathway enrichment analysis for the curated list of cancer beating molecules (CBM) from FooDB with anti-cancer likeness >0.7

References

1 Molnar, J. *et al.* Investigation of the Antiproliferative Properties of Natural Sesquiterpenes from Artemisia asiatica and Onopordum acanthium on HL-60 Cells in Vitro. *Int J Mol Sci* **17**, 83, doi:10.3390/ijms17020083 (2016).

2 Zhang, S., Won, Y. K., Ong, C. N. & Shen, H. M. Anti-cancer potential of sesquiterpene lactones: bioactivity and molecular mechanisms. *Curr Med Chem Anticancer Agents* **5**, 239-249 (2005).

3 Pommier, Y. Topoisomerase I inhibitors: camptothecins and beyond. *Nat Rev Cancer* **6**, 789-802, doi:10.1038/nrc1977 (2006).

4 McNeil, C. Topotecan: after FDA and ASCO, what's next? *J Natl Cancer Inst* **88**, 788-789 (1996).

5 Saleem, M. Lupeol, a novel anti-inflammatory and anti-cancer dietary triterpene. *Cancer Lett* **285**, 109-115, doi:10.1016/j.canlet.2009.04.033 (2009).

6 Chaturvedi, P. K., Bhui, K. & Shukla, Y. Lupeol: connotations for chemoprevention. *Cancer Lett* **263**, 1-13, doi:10.1016/j.canlet.2008.01.047 (2008).

7 Lee, Y. Cancer Chemopreventive Potential of Procyanidin. *Toxicol Res* **33**, 273-282, doi:10.5487/TR.2017.33.4.273 (2017).

8 Kaschula, C. H., Hunter, R. & Parker, M. I. Garlic-derived anticancer agents: structure and biological activity of ajoene. *Biofactors* **36**, 78-85, doi:10.1002/biof.76 (2010).

9 Li, M. *et al.* Antitumor activity of Z-ajoene, a natural compound purified from garlic: antimitotic and microtubule-interaction properties. *Carcinogenesis* **23**, 573-579 (2002).

10 Wei, P. L. *et al.* The in vivo antitumor effects on human COLO 205 cancer cells of the 4,7-dimethoxy-5-(2-propen-1-yl)-1,3-benzodioxole (apiole) derivative of 5-substituted 4,7-dimethoxy-5-methyl-l,3-benzodioxole (SY-1) isolated from the fruiting body of Antrodia camphorate. *J Cancer Res Ther* **8**, 532-536, doi:10.4103/0973-1482.106529 (2012).

11 Alghasham, A. A. Cucurbitacins - a promising target for cancer therapy. *Int J Health Sci (Qassim)* **7**, 77-89 (2013).

12 Militao, G. C. *et al.* In vitro and in vivo anticancer properties of cucurbitacin isolated from Cayaponia racemosa. *Pharm Biol* **50**, 1479-1487, doi:10.3109/13880209.2012.684691 (2012).

13 Singhal, S. S. *et al.* Didymin: an orally active citrus flavonoid for targeting neuroblastoma. *Oncotarget* **8**, 29428-29441, doi:10.18632/oncotarget.15204 (2017).

14 Yao, Q., Lin, M. T., Zhu, Y. D., Xu, H. L. & Zhao, Y. Z. Recent Trends in Potential Therapeutic Applications of the Dietary Flavonoid Didymin. *Molecules* **23**, doi:10.3390/molecules23102547 (2018).

15 Luo, M. *et al.* Cajanol, a novel anticancer agent from Pigeonpea [Cajanus cajan (L.) Millsp.] roots, induces apoptosis in human breast cancer cells through a ROS-mediated mitochondrial pathway. *Chem Biol Interact* **188**, 151-160, doi:10.1016/j.cbi.2010.07.009 (2010).

16 Liang, L. *et al.* The phytoestrogenic compound cajanol from Pigeonpea roots is associated with the activation of estrogen receptor alpha-dependent signaling

pathway in human prostate cancer cells. *Phytother Res* **27**, 1834-1841, doi:10.1002/ptr.4937 (2013).
17 Park, K. C., Kim, S. Y. & Kim, D. S. Experimental photodynamic therapy for liver cancer cell-implanted nude mice by an indole-3-acetic acid and intense pulsed light combination. *Biol Pharm Bull* **32**, 1609-1613 (2009).
18 Kim, S. Y. *et al.* UVB-activated indole-3-acetic acid induces apoptosis of PC-3 prostate cancer cells. *Anticancer Res* **30**, 4607-4612 (2010).
19 Thippeswamy, G., Sheela, M. L. & Salimath, B. P. Octacosanol isolated from Tinospora cordifolia downregulates VEGF gene expression by inhibiting nuclear translocation of NF-<kappa>B and its DNA binding activity. *Eur J Pharmacol* **588**, 141-150, doi:10.1016/j.ejphar.2008.04.027 (2008).
20 Huber, W. W. *et al.* Potential chemoprotective effects of the coffee components kahweol and cafestol palmitates via modification of hepatic N-acetyltransferase and glutathione S-transferase activities. *Environ Mol Mutagen* **44**, 265-276, doi:10.1002/em.20052 (2004).
21 Lima, C. S. *et al.* Cafestol, a diterpene molecule found in coffee, induces leukemia cell death. *Biomed Pharmacother* **92**, 1045-1054, doi:10.1016/j.biopha.2017.05.109 (2017).
22 de Oliveira, P. F., Munari, C. C., Nicolella, H. D., Veneziani, R. C. & Tavares, D. C. Manool, a Salvia officinalis diterpene, induces selective cytotoxicity in cancer cells. *Cytotechnology* **68**, 2139-2143, doi:10.1007/s10616-015-9927-0 (2016).
23 Kim, Y. S. & Milner, J. A. Targets for indole-3-carbinol in cancer prevention. *J Nutr Biochem* **16**, 65-73, doi:10.1016/j.jnutbio.2004.10.007 (2005).
24 Weng, J. R., Tsai, C. H., Kulp, S. K. & Chen, C. S. Indole-3-carbinol as a chemopreventive and anti-cancer agent. *Cancer Lett* **262**, 153-163, doi:10.1016/j.canlet.2008.01.033 (2008).
25 Boyd, J. & Han, A. Deguelin and Its Role in Chronic Diseases. *Adv Exp Med Biol* **929**, 363-375, doi:10.1007/978-3-319-41342-6_16 (2016).
26 Baba, Y. & Kato, Y. Deguelin, a Novel Anti-Tumorigenic Agent in Human Esophageal Squamous Cell Carcinoma. *EBioMedicine* **26**, 10, doi:10.1016/j.ebiom.2017.11.010 (2017).
27 Wu, Q. P. *et al.* Ergosterol peroxide isolated from Ganoderma lucidum abolishes microRNA miR-378-mediated tumor cells on chemoresistance. *PLoS One* **7**, e44579, doi:10.1371/journal.pone.0044579 (2012).
28 Wu, H. Y. *et al.* Ergosterol peroxide from marine fungus Phoma sp. induces ROS-dependent apoptosis and autophagy in human lung adenocarcinoma cells. *Sci Rep* **8**, 17956, doi:10.1038/s41598-018-36411-2 (2018).
29 Lee, S. E. & Lee, J. K. Sesamolin affects both natural killer cells and cancer cells in order to create an optimal environment for cancer cell sensitization. *Int Immunopharmacol* **64**, 16-23, doi:10.1016/j.intimp.2018.08.027 (2018).
30 Jiang, W. L., Zhang, S. P., Zhu, H. B., Jian, H. & Tian, J. W. Cornin ameliorates cerebral infarction in rats by antioxidant action and stabilization of mitochondrial function. *Phytother Res* **24**, 547-552, doi:10.1002/ptr.2978 (2010).
31 Yuan, Y. *et al.* Shufeng Jiedu Capsules Alleviate Lipopolysaccharide-Induced Acute Lung Inflammatory Injury via Activation of GPR18 by Verbenalin. *Cell Physiol Biochem* **50**, 629-639, doi:10.1159/000494184 (2018).

32 Kong, C. S., Kim, J. A., Yoon, N. Y. & Kim, S. K. Induction of apoptosis by phloroglucinol derivative from Ecklonia Cava in MCF-7 human breast cancer cells. *Food Chem Toxicol* **47**, 1653-1658, doi:10.1016/j.fct.2009.04.013 (2009).

33 Kim, R. K. *et al.* Novel anticancer activity of phloroglucinol against breast cancer stem-like cells. *Toxicol Appl Pharmacol* **286**, 143-150, doi:10.1016/j.taap.2015.03.026 (2015).

34 Hsu, Y. L., Hou, M. F., Tsai, E. M. & Kuo, P. L. Tricetin, a dietary flavonoid, induces apoptosis through the reactive oxygen species/c-Jun NH2-terminal kinase pathway in human liver cancer cells. *J Agric Food Chem* **58**, 12547-12556, doi:10.1021/jf103159r (2010).

35 Chang, P. Y. *et al.* Tricetin inhibits human osteosarcoma cells metastasis by transcriptionally repressing MMP-9 via p38 and Akt pathways. *Environ Toxicol* **32**, 2032-2040, doi:10.1002/tox.22380 (2017).

36 Tang, H. *et al.* Anti-colon cancer effect of caffeic acid p-nitro-phenethyl ester in vitro and in vivo and detection of its metabolites. *Sci Rep* **7**, 7599, doi:10.1038/s41598-017-07953-8 (2017).

37 Pelinson, L. P. *et al.* Antiproliferative and apoptotic effects of caffeic acid on SK-Mel-28 human melanoma cancer cells. *Mol Biol Rep*, doi:10.1007/s11033-019-04658-1 (2019).

38 Serafim, T. L. *et al.* Lipophilic caffeic and ferulic acid derivatives presenting cytotoxicity against human breast cancer cells. *Chem Res Toxicol* **24**, 763-774, doi:10.1021/tx200126r (2011).

39 Gao, J. *et al.* The anticancer effects of ferulic acid is associated with induction of cell cycle arrest and autophagy in cervical cancer cells. *Cancer Cell Int* **18**, 102, doi:10.1186/s12935-018-0595-y (2018).

40 Paduch, R. *et al.* Biological activity of terpene compounds produced by biotechnological methods. *Pharm Biol* **54**, 1096-1107, doi:10.3109/13880209.2015.1103753 (2016).

41 Tanaka, T. *et al.* Citrus limonoids obacunone and limonin inhibit azoxymethane-induced colon carcinogenesis in rats. *Biofactors* **13**, 213-218 (2000).

42 Kim, J., Jayaprakasha, G. K. & Patil, B. S. Obacunone exhibits anti-proliferative and anti-aromatase activity in vitro by inhibiting the p38 MAPK signaling pathway in MCF-7 human breast adenocarcinoma cells. *Biochimie* **105**, 36-44, doi:10.1016/j.biochi.2014.06.002 (2014).

43 Kalalinia, F. & Karimi-Sani, I. Anticancer Properties of Solamargine: A Systematic Review. *Phytother Res* **31**, 858-870, doi:10.1002/ptr.5809 (2017).

44 Steigerova, J. *et al.* Mechanisms of natural brassinosteroid-induced apoptosis of prostate cancer cells. *Food Chem Toxicol* **50**, 4068-4076, doi:10.1016/j.fct.2012.08.031 (2012).

45 Sadava, D. & Kane, S. E. The effect of brassinolide, a plant steroid hormone, on drug resistant small-cell lung carcinoma cells. *Biochem Biophys Res Commun* **493**, 783-787, doi:10.1016/j.bbrc.2017.08.094 (2017).

46 Gompel, A. & Plu-Bureau, G. Ovarian cancer and hormone replacement therapy. *Lancet* **370**, 932; author reply 932-933, doi:10.1016/S0140-6736(07)61436-6 (2007).

47 Jerry, D. J. Roles for estrogen and progesterone in breast cancer prevention. *Breast Cancer Res* **9**, 102, doi:10.1186/bcr1659 (2007).
48 Ni, X., Zhang, A., Zhao, Z., Shen, Y. & Wang, S. Demethoxycurcumin inhibits cell proliferation, migration and invasion in prostate cancer cells. *Oncol Rep* **28**, 85-90, doi:10.3892/or.2012.1783 (2012).
49 Lin, C. C. *et al.* Demethoxycurcumin Suppresses Migration and Invasion of Human Cervical Cancer HeLa Cells via Inhibition of NF-kappaB Pathways. *Anticancer Res* **38**, 2761-2769, doi:10.21873/anticanres.12519 (2018).
50 Fu, Y. *et al.* Cell cycle arrest and induction of apoptosis by cajanin stilbene acid from Cajanus cajan in breast cancer cells. *Phytomedicine* **22**, 462-468, doi:10.1016/j.phymed.2015.02.005 (2015).
51 Fu, Y. *et al.* Activity of the antiestrogenic cajanin stilbene acid towards breast cancer. *J Nutr Biochem* **26**, 1273-1282, doi:10.1016/j.jnutbio.2015.06.004 (2015).
52 Potze, L., Mullauer, F. B., Colak, S., Kessler, J. H. & Medema, J. P. Betulinic acid-induced mitochondria-dependent cell death is counterbalanced by an autophagic salvage response. *Cell Death Dis* **5**, e1169, doi:10.1038/cddis.2014.139 (2014).
53 Fulda, S. Betulinic Acid for cancer treatment and prevention. *Int J Mol Sci* **9**, 1096-1107, doi:10.3390/ijms9061096 (2008).
54 Chen, Q. F., Liu, Z. P. & Wang, F. P. Natural sesquiterpenoids as cytotoxic anticancer agents. *Mini Rev Med Chem* **11**, 1153-1164 (2011).
55 Zheng, G. Q., Kenney, P. M. & Lam, L. K. Anethofuran, carvone, and limonene: potential cancer chemopreventive agents from dill weed oil and caraway oil. *Planta Med* **58**, 338-341, doi:10.1055/s-2006-961480 (1992).
56 Patel, P. B. & Thakkar, V. R. L-carvone induces p53, caspase 3 mediated apoptosis and inhibits the migration of breast cancer cell lines. *Nutr Cancer* **66**, 453-462, doi:10.1080/01635581.2014.884230 (2014).
57 Kannappan, R., Gupta, S. C., Kim, J. H. & Aggarwal, B. B. Tocotrienols fight cancer by targeting multiple cell signaling pathways. *Genes Nutr* **7**, 43-52, doi:10.1007/s12263-011-0220-3 (2012).
58 Sertel, S., Eichhorn, T., Plinkert, P. K. & Efferth, T. Chemical Composition and antiproliferative activity of essential oil from the leaves of a medicinal herb, Levisticum officinale, against UMSCC1 head and neck squamous carcinoma cells. *Anticancer Res* **31**, 185-191 (2011).
59 Mehmood, T., Maryam, A., Tian, X., Khan, M. & Ma, T. Santamarine Inhibits NF-small ka, CyrillicB and STAT3 Activation and Induces Apoptosis in HepG2 Liver Cancer Cells via Oxidative Stress. *J Cancer* **8**, 3707-3717, doi:10.7150/jca.20239 (2017).
60 Ma, G., Chong, L., Li, Z., Cheung, A. H. & Tattersall, M. H. Anticancer activities of sesquiterpene lactones from Cyathocline purpurea in vitro. *Cancer Chemother Pharmacol* **64**, 143-152, doi:10.1007/s00280-008-0863-y (2009).
61 Wu, C. H., Hong, B. H., Ho, C. T. & Yen, G. C. Targeting cancer stem cells in breast cancer: potential anticancer properties of 6-shogaol and pterostilbene. *J Agric Food Chem* **63**, 2432-2441, doi:10.1021/acs.jafc.5b00002 (2015).

62 Nedungadi, D. *et al.* 6-Shogaol induces caspase-independent paraptosis in cancer cells via proteasomal inhibition. *Exp Cell Res* **364**, 243-251, doi:10.1016/j.yexcr.2018.02.018 (2018).
63 Sethi, G. *et al.* Pro-Apoptotic and Anti-Cancer Properties of Diosgenin: A Comprehensive and Critical Review. *Nutrients* **10**, doi:10.3390/nu10050645 (2018).
64 Iranshahi, M., Sahebkar, A., Takasaki, M., Konoshima, T. & Tokuda, H. Cancer chemopreventive activity of the prenylated coumarin, umbelliprenin, in vivo. *Eur J Cancer Prev* **18**, 412-415, doi:10.1097/CEJ.0b013e32832c389e (2009).
65 Rashidi, M. *et al.* Umbelliprenin shows antitumor, antiangiogenesis, antimetastatic, anti-inflammatory, and immunostimulatory activities in 4T1 tumor-bearing Balb/c mice. *J Cell Physiol* **233**, 8908-8918, doi:10.1002/jcp.26814 (2018).
66 Elegbede, J. A., Elson, C. E., Qureshi, A., Tanner, M. A. & Gould, M. N. Inhibition of DMBA-induced mammary cancer by the monoterpene d-limonene. *Carcinogenesis* **5**, 661-664 (1984).
67 Yu, X. *et al.* d-limonene exhibits antitumor activity by inducing autophagy and apoptosis in lung cancer. *Onco Targets Ther* **11**, 1833-1847, doi:10.2147/OTT.S155716 (2018).
68 Hassan, S. B., Gali-Muhtasib, H., Goransson, H. & Larsson, R. Alpha terpineol: a potential anticancer agent which acts through suppressing NF-kappaB signalling. *Anticancer Res* **30**, 1911-1919 (2010).
